# Robo2 Receptor Gates the Anatomical Divergence of Neurons Derived From a Common Precursor Origin

**DOI:** 10.3389/fcell.2021.668175

**Published:** 2021-06-23

**Authors:** Maud Wurmser, Mridula Muppavarapu, Christine Mary Tait, Christophe Laumonnerie, Luz María González-Castrillón, Sara Ivy Wilson

**Affiliations:** ^1^Department of Integrative Medical Biology, Umeå University, Umeå, Sweden; ^2^Umeå University, Umeå, Sweden

**Keywords:** migration, axon guidance, robo receptors, neural development, commissural neuron, ipsilateral neuron, neural organization, sensory neuron

## Abstract

Sensory information relayed to the brain is dependent on complex, yet precise spatial organization of neurons. This anatomical complexity is generated during development from a surprisingly small number of neural stem cell domains. This raises the question of how neurons derived from a common precursor domain respond uniquely to their environment to elaborate correct spatial organization and connectivity. We addressed this question by exploiting genetically labeled mouse embryonic dorsal interneuron 1 (dI1) neurons that are derived from a common precursor domain and give rise to spinal projection neurons with distinct organization of cell bodies with axons projecting either commissurally (dI1c) or ipsilaterally (dI1i). In this study, we examined how the guidance receptor, Robo2, which is a canonical Robo receptor, influenced dI1 guidance during embryonic development. Robo2 was enriched in embryonic dI1i neurons, and loss of *Robo2* resulted in misguidance of dI1i axons, whereas dI1c axons remained unperturbed within the mantle zone and ventral commissure. Further, Robo2 profoundly influenced dI1 cell body migration, a feature that was partly dependent on Slit2 signaling. These data suggest that dI1 neurons are dependent on Robo2 for their organization. This work integrated with the field support of a model whereby canonical Robo2 vs. non-canonical Robo3 receptor expression facilitates projection neurons derived from a common precursor domain to read out the tissue environment uniquely giving rise to correct anatomical organization.

## Introduction

The physiological function of the nervous system is dependent on the precise spatial connectivity of a diverse range of neural populations. This mature spatial organization originates from a relatively small number of progenitor domains, raising the broad question of how neurons derived from a common precursor origin and environment elaborate the spatial organization required for their later functional connectivity.

During development, patterned multipotent neural stem cells differentially express key transcription factors instructing a cascade of events leading to their differentiation. Developing neurons subsequently migrate and grow in response to cues in their environment in a subtype-specific manner. This is achieved by the differential expression and localization of a small number of ligand/receptor molecular pathways used in various combinatorial codes together with various adapter molecules and gating signaling molecules to elicit specific responses. These broadly evolutionarily conserved pathways include the Slit/Robo, Eph/Ephrin, Semaphorin/plexin, neuropilin and Netrin/DCC, Uncs signaling, in addition to growth factors (Stoeckli, 2018). Of these, Robo signaling is an indispensable regulator of neurodevelopment and is linked to a number of human disorders including cancer, neurodevelopmental disorders such as schizophrenia, dyslexia, and horizontal gaze palsy with progressive scoliosis as well as other fundamental biological processes (Blockus and Chedotal, 2016; Gonda et al., 2020). Robo receptors are transmembrane proteins that interact with Slit ligands and other molecules to elicit signaling responses (Zelina et al., 2014; Jaworski et al., 2015; Blockus and Chedotal, 2016; Taroc et al., 2019; Gonda et al., 2020). In a wide range of species, Robo signaling is known to be imperative in the neuronal guidance of a range of neurons including implementing organization of longitudinally projecting axons within the white matter tracts and in commissural axon guidance. Classically in this context, Robo receptors convey repellent signaling, important for regulating commissural axon crossing and exit from the ventral commissure and positioning of commissural and ipsilateral axons tracts within the white matter in addition to cell migration processes (Kidd et al., 1998; Brose et al., 1999; Long et al., 2004; Nguyen-Ba-Charvet et al., 2004; Devine and Key, 2008; Farmer et al., 2008; Geisen et al., 2008; Jaworski et al., 2010; Neuhaus-Follini and Bashaw, 2015; Blockus and Chedotal, 2016; Ducuing et al., 2019; Gruner et al., 2019; Johnson et al., 2019). For simplicity of description in this paper, this “classical” Robo-repellent feature is referred to as “canonical” Robo function. Given this, a surprising discovery was that in mammals, a splice variant of Robo3, Robo3.1, has been shown to have the “opposite” or “non-canonical” function in the guidance of commissural axons of the caudal nervous system in mice. In humans, Robo3 gene deficits result in corresponding commissural axon anatomical defects and horizontal gaze palsy with progressive scoliosis (Jen et al., 2004; Marillat et al., 2004; Sabatier et al., 2004; Friocourt and Chedotal, 2017).

**FIGURE 1:**
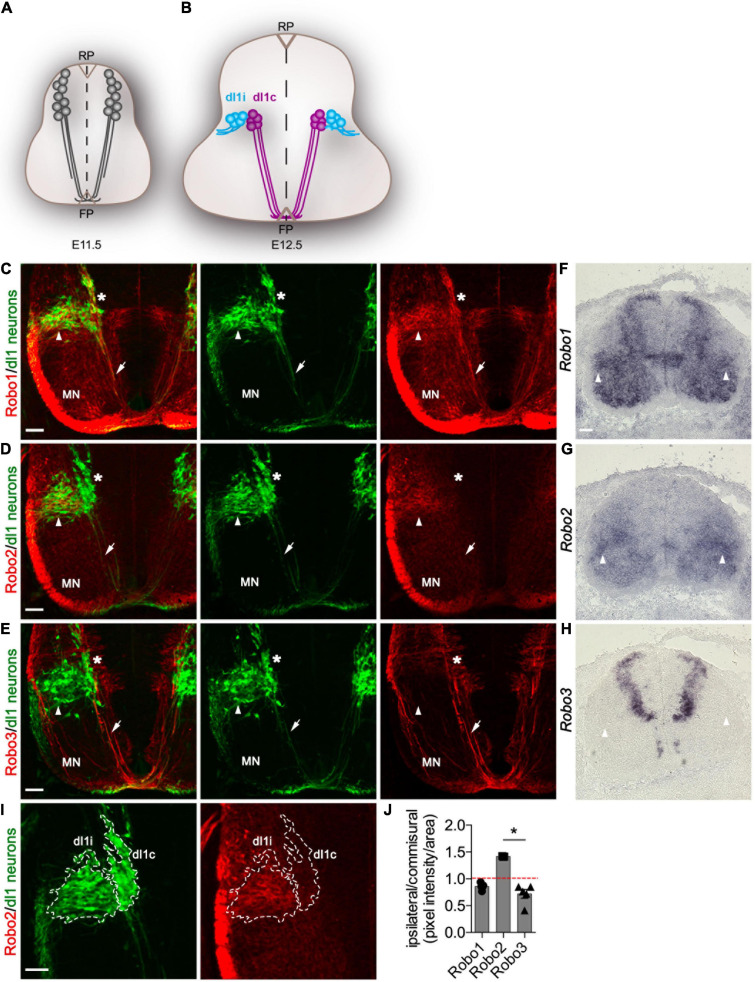
Robo2 but not Robo3 is enriched in di1 ipsilateral neurons. **(A,B)** Schematic representation of a transverse section of the spinal cord illustrating the expression of the *Barhl2^*GFP*^* transgene used in this study to genetically delineate dI1 neurons (gray) at E11.5 **(A)** and E12. **(B)** dI1 commissural (dI1c) and dI1 ipsilateral (dI1i) cell body and axonal projections are depicted. **(C–E)** Photomicrographs of *Barhl2^*GFP*^* E12.5 mouse embryonic spinal cord brachial transverse sections. The images show immunohistochemical labeling with GFP (green) to delineate dI1 neurons and with Robo1 (red) **(C)**, Robo2 (red) **(D)**, or Robo3 (red) **(E)**, respectively. The single-channel GFP images and the Robo1/GFP, Robo2/GFP and Robo3/GFP merged image are shown. The white arrow points to commissural axons and the arrowhead to dI1i axons and soma. The asterisk * indicates the dorsoventral position of dI1c cell bodies. The position of motor neurons (MN) is indicated. At least three embryos were analyzed for each condition. Representative images are shown. **(F–H)** Photomicrographs of brachial spinal cord sections from E12.5 *Barhl2^*GFP*^* embryos labeled by *in situ* hybridization for *Robo1* **(F)** *Robo2* **(G)** or *Robo3* **(H).** At least three embryos were analyzed for each condition. **(I)** Example photomicrographs of the quantification method used illustrated for the sample shown in **C**. Brachial E12.5 spinal cord samples were labeled with GFP and Robo1, Robo2, or Robo3. The GFP labeling used to anatomically define dI1c or dI1i neurons (outlined in I). **(J)** Quantification of the relative expression of Robo1 (*n* = 5), Robo2 (*n* = 3), and Robo3 (*n* = 5) expressed as a ratio of labeling in dI1c vs. dI1i neurons. Mean, standard errors, and statistical significance are shown. One way ANOVA Kruskal–Wallis test followed by Dunn’s multiple-comparison analysis was performed. Scale bars in **(C–I)** are 50 μm and represent the images within the same panel.

Overall, good progress has been made in understanding how Robo signaling and other molecular pathways promote correct guidance and organization of individual classes of neurons. However, in mammals the molecular logic of how this is gated by neurons derived from a common precursor domain or more broadly from multipotent stem cells remains poorly understood. The phenotypic antagonism between canonical vs. non-canonical Robo signaling could serve as a potential mammalian evolved mechanism to drive anatomical diversity from a common precursor domain; however, this remains to be examined. In two of the major systems used to probe neural diversification, the cortex, and the retina, non-canonical Robo3 signaling does not appear to play a major role in commissural axon formation (Jen et al., 2004; Lodato and Arlotta, 2015; Friocourt and Chedotal, 2017; Mason and Slavi, 2020). Therefore, here we have taken advantage of a transgenic mouse model, which genetically labels embryonic spinal cord neurons derived from a common precursor origin, precursor dorsal interneuron 1 (pdI1), which give rise to both commissural and ipsilateral projecting dorsal interneuron 1 (dI1c and dI1i, respectively) neurons (Figures 1A,B; Wilson et al., 2008). This provides an ideal model system to examine how individual subsets of neurons derived from a common precursor origin respond uniquely to the tissue environment to elaborate spatial organization. We discovered that Robo2 receptor expression was enriched in dI1i neurons within the gray matter and that knocking out the *Robo2* gene in mice resulted in misguidance of dI1 cell bodies and axons within the embryonic mantle zone. We further found that the misprojecting axons in *Robo2* mutant embryos were dI1i neurons whereas pre-crossing and crossing dI1c neurons appeared unperturbed. This *Robo2* phenotype was marginally enhanced by the loss of *Robo1* and was partially phenocopied by the loss of *Slit2*. This indicated that Robo1 and Robo2 were not redundant in this context and that this phenotypic effect was elaborated at least in part through canonical Slit/Robo signaling. Overall, these findings taken together with previous results showing that non-canonical Robo3 controls dI1c axon guidance reveal a mechanism whereby Robo2 and Robo3 are differentially enriched at a key anatomical divergence point and result in the ability of dI1i and dI1c neurons derived from a common precursor origin to respond uniquely to their environment to elaborate the neural organization needed for their latter function.

## Materials and Methods

### Mice and Genotyping

The following mice were used: *Wild type* SvEv129, *Barhl2^*GFP*^*, *Math1^*LACZ*^*, *Robo1^+/–^:Robo2^+/–^*, *Robo2*^+/–^, and *Slit2*^+/–^ mice (Helms and Johnson, 1998; Grieshammer et al., 2004; Long et al., 2004; Wilson et al., 2008). Of these, the *Robo2*^+/–^ line was derived from the *Robo1^+/–^:Robo2^+/–^* line by a natural linkage brake. The genetically modified mice were maintained in a mixed genetic background, composed combination of SvEv129, Swiss Webster, and NMRI lines. Mice were genotyped as previously described (Helms and Johnson, 1998; Grieshammer et al., 2004; Long et al., 2004; Wilson et al., 2008).

### Embryo Processing

Staged embryos were obtained and processed as previously described (Laumonnerie et al., 2015). Samples were excluded if they were clearly morphologically young or the sample was sectioned in a way that did not permit accurate analysis regardless of genotype. The sex of the samples was not tracked.

### *In Situ* Hybridization

*In situ* hybridization was performed as previously described using probes against mouse *Robo1*, *Robo2*, *Robo3*, and rat *Slit1*, *Slit2*, and *Slit3* (Brose et al., 1999; Wilson et al., 2008; Carr et al., 2017).

### Immunohistochemistry

Antibodies produced in this paper, rabbit α-Lhx2, guinea pig α-Lhx9, rabbit α-Robo1, guinea pig α-Robo2, and rabbit α-Robo3, are described and characterized in the Supplementary Data. Immunohistochemistry was performed as described previously (Kropp and Wilson, 2012; Laumonnerie et al., 2014, 2015) with the exception of α-Robo2 antibodies, which were incubated overnight at 30°C in blocking buffer containing 5% FBS, 0.1% Triton X-100 in 1 × PBS (77 mM Na_2_HPO_4_, 23 mM NaH_2_PO_4_, and 1.5 M NaCl). The following primary antibodies were used: chicken α-GFP (1/1000 Aves Labs, GFP-1020), chicken α-GFP (ABCAM, ab13970), rabbit α-Robo1 antibody produced in this paper (used in the quantification and analysis), rabbit α-Robo1 (Tamada et al., 2008), guinea pig α-Robo2 antibody produced in this paper (used in the quantification and analysis), rabbit α-Robo2 (Tamada et al., 2008), goat α-Robo2 (1/100 R and D Systems, AF3147), rabbit α-Robo3 antibody produced in this paper (used for the quantification and analysis), rabbit α-Lhx2 produced in this paper (1/2,000–1/32,000), rabbit α-Lhx9 produced in this paper (1/2,000), rabbit α-Barhl2 (1/500), rabbit α-Robo3 (Tamada et al., 2008), goat α-Robo3 (1/500 R and D Systems, AF3076), and goat α-ß-galactosidase (1/2,000 Biogenics). The following secondary antibodies were used: goat α-chicken FITC (F-1005) from Aves Labs, OR, United States; goat α-guinea pig Cy3 (106-165-003); donkey α-chicken FITC (c703-096-155); donkey α-rabbit Cy3 (711-165-152); and donkey α-goat Cy3 (705-165-003) from Jackson ImmunoResearch Europe Ltd.

### Imaging and Image Processing

**FIGURE 2:**
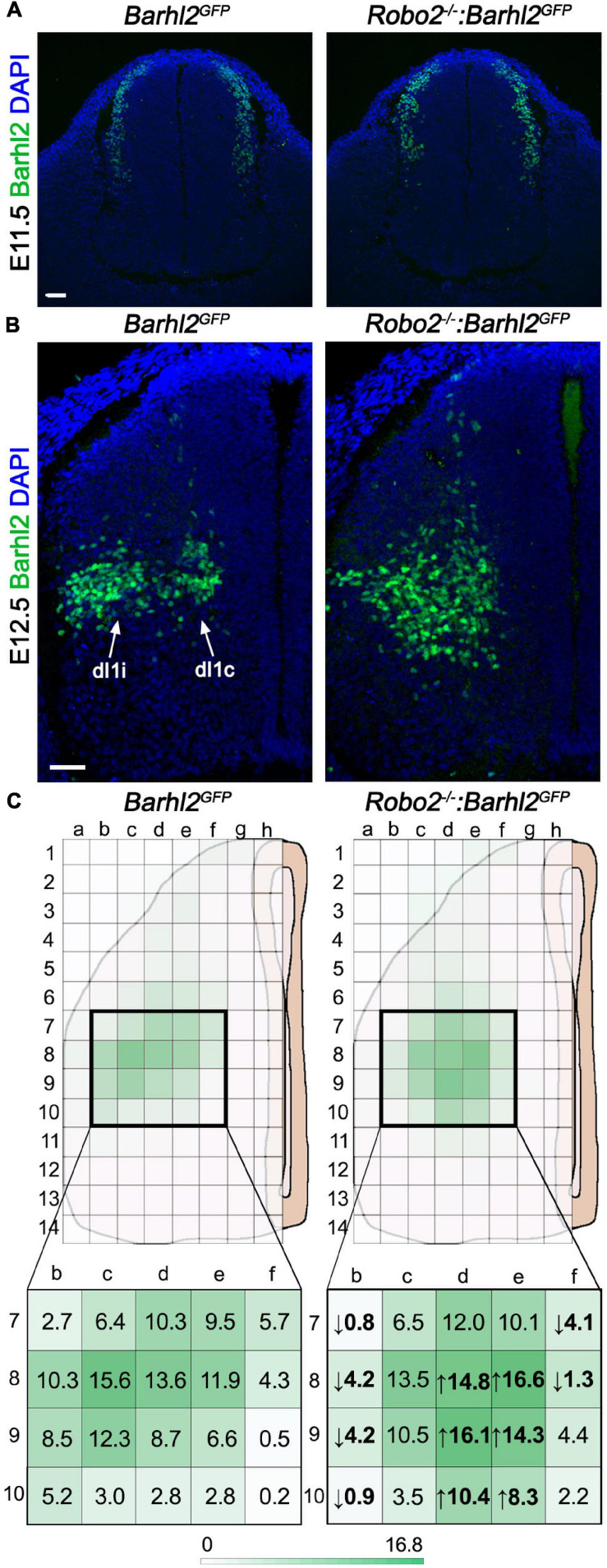
Quantification of di1 cell body migration in *Robo2^–/–^:Barhl2^*GFP*^* and control embryos. **(A,B)** Example photomicrographs of mouse embryonic spinal cord tissue at E11.5 **(A)** and E12.5 **(B)** immunohistochemically labeled with the dI1 transcription factor Barhl2 in control *Barhl2^*GFP*^* (E11.5 *n* = 5, E12.5 *n* = 6) and *Robo2* mutant *Robo2^–/–^:Barhl2^*GFP*^* (E11.5 *n* = 3, E12.5 *n* = 8) embryos. **(C)** Schematics representing an E12.5 spinal cord hemisection with the overlying grid used for demarking bins for counting Barhl2^+^ nuclei for quantification are depicted. The mean number of Barhl2^+^ cells shown for control (*n* = 6) and in *Robo2^–/–^* (*n* = 8) is depicted by a heat map (green). The bold boxed regions are enlarged to show the mean number of Barhl2^+^ neurons per bin. Values in bold are the statistically significant increase (up arrow) or decrease (down arrow), after two-way ANOVA statistical test. Each individual box mean and statistical significance are shown in Supplementary Table 1. Scale bar in **(A,B)** are 50 μm and represents images within the same panel.

Samples were imaged using a Nikon Eclipse E800 or Leica DM6000B fluorescence microscope and Zeiss LSM 710 or LSM 510 and Leica SP8 Falcon confocal microscope. Figures were assembled in Adobe Photoshop CS4. Images presented in the figures are raw data images processed for orientation and cropped to size and pixel density with the following exception: In Figure 2 and Supplementary Figures 9, 11, the image brightness was increased to better visualize the data.

### Experimental Design, Number of Samples, Statistics, and Quantification

The number of samples used in each experiment is indicated in the figure legends. For the experiments in this paper with the exception of the quantification and the Lhx2 and Lhx9 transfections in Supplementary Figure 6, which is detailed below and in Supplementary Figure 6 figure legend, the number of times experiments were repeated was at least 3, which is the standard in the field. For each experiment involving embryos, different individual embryos were analyzed and different sections within the same embryo were also analyzed. The number of individual embryos for each specific experiment, condition, and type of analysis was between 3 and > 8, as indicated in the respective figure legend. This difference reflects if the analysis was highly stereotyped (e.g., antibody labeling) or where variation was observed (e.g., phenotype analysis) and whether statistical analysis was performed. The number of individual embryos selected was standard or greater than is used in the field. The sex of the embryos used in this study was not tracked and therefore was randomized. Experiments where statistical analysis was performed with 3 to 10 embryos per genotype are detailed below. This is more than is typical in the field when using mouse embryos and provided a good representation of the range of phenotype observed. A detailed description of the quantification and statistical analysis is in the respective method sections. Since the phenotypes observed were clear, it was not possible to truly blind all the analyses although blinding has been performed during quantification analysis in that the cell counting was performed with the experimentalist blind to the genotype.

### Quantification of Robo1, Robo2, and Robo3 Expression

E12.5 spinal cord tissue from *Barhl2* transgenic embryos were immunohistochemically labeled with GFP and either Robo1, Robo2, or Robo3 (*n* = 5, 3, and 5 embryos, respectively). Z-stack photomicrographs (20×) were acquired using the Leica SP8 Falcon confocal microscope. Maximum projection images were generated using ImageJ, and the region of interest (ROI) was traced to demarcate dl1i and dl1c populations based on anatomy. The ratio between dl1i/dl1c of Robo1, Robo2, or Robo3 pixel intensity was quantified. Statistical analysis was performed as follows: Using Prism software, a one-way ANOVA Kruskal–Wallis non-parametric test followed by Dunn’s multiple-comparison analysis was performed.

### Quantification of Neural Migration Phenotype

For quantification of the Barhl2^+^ neuron migration phenotype, transverse brachial sections of E12.5 *Robo2^+/+^:Barhl2^*GFP*^* (control, *n* = 6), *Robo2^+/–^:Barhl2^*GFP*^* (heterozygote, *n* = 7), *Robo2^–/–^:Barhl2^*GFP*^* (mutant, *n* = 8), Slit*2^+/+^:Math1^*LacZ*^* (control, *n* = 5), and Slit*2^–/–^:Math1^*LacZ*^* (mutant, *n* = 6), *Robo1^+/+^:Robo2^+/+^:Barhl2^*GFP*^* (control, *n* = 7), *Robo1^–/+^:Robo2^–/+^:Barhl2^*GFP*^* (heterozygous, *n* = 7), and *Robo1^–/–^:Robo2^–/–^:Barhl2^*GFP*^* (mutant, *n* = 7) embryos were immunohistochemically labeled with Barhl2 to label dI1 neurons. From the images collected, a grid of 8 times 14 bins was superimposed on the images (Photoshop) so that the grid fitted the dorso-ventral and medial-lateral extreme of each spinal cord hemi-section analyzed (example in Figure 2). This created a grid composed of 112 binds. The number of Barhl2^+^ nuclei was counted for each bin and expressed as a mean in Figure 2 and Supplementary Figures 9, 11. The raw data counts are shown in Supplementary Tables 1–3. For bins where the mean cell count was greater than 3, a Shapiro normality test was applied using R software. By this measure, greater than 80% of the bins showed a normal distribution and therefore a two-way ANOVA followed by Sidak’s multiple-comparison test was performed using Prism software.

For the quantification of the Lhx9^+^ neural migration phenotype, transverse thoracic sections of E12.5 *Robo2^+/+^:Barhl2^*GFP*^* (control, *n* = 7) and *Robo2^–/–^:Barhl2^*GFP*^* (mutant, *n* = 10) embryos were immunohistochemically labeled with the Lhx9 antibody. From the images collected, the dorso-ventral position of the main cohort of Lhx9^+^ neurons was measured, relative to the dorso-ventral length of the spinal cord in the section being examined. This percentage value was then averaged for control embryos which establish an average position of the control Lhx9^+^ neuron. This was 38% for control embryos (*n* = 7). A 38% ventral cutoff line was positioned on all the spinal cord images of control and mutant embryos (see red dotted line, Supplementary Figure 7B), and the number of Lhx9^+^ neurons located ventral to this cutoff line was counted from both sides of the spinal cord and averaged. This analysis was performed with the experimenter blinded to the genotype of the samples. All measures were performed using ImageJ software. The average number of cells located ventrally from the cutoff line was compared between groups using a Mann–Whitney statistical analysis using Prism software.

### Quantification of GFP^+^ Commissural Axons in Mice Carrying the *Barhl2^*GFP*^* Transgene

#### Ventral Commissure GFP^+^ Axon Quantification

Transverse brachial sections of 11.5 and E12.5 *Robo2^+/+^:Barhl2^*GFP*^* (control, E11.5 *n* = 5, E12.5 *n* = 9), *Robo2^+/–^:Barhl2^*GFP*^* (heterozygote, E11.5 *n* = 6, E12.5 *n* = 9), and *Robo2^–/–^:Barhl2^*GFP*^* (mutant, E11.5 *n* = 5, E12.5 *n* = 10) embryos were immunohistochemically labeled with Robo3 to label commissural axons and GFP to label dI1 neurons and were imaged with a Leica SP8 Falcon confocal microscope. Quantification was performed on the maximal projection of Z-stack images. Within ImageJ, two fixed-size boxes were applied to each image, Box 2 within the ventral mantle zone and Box 1 within the ventral commissure (Figure 4). The pixel intensity was measured within the boxes, normalized to the boxes’ area, and a ratio of Box1/Box2 was calculated for each image. For each genotype, one section for each embryo was measured. The measurements for each genotype were collated and statistical analysis included Kruskal–Wallis non-parametric tests followed by Dunn’s multiple-comparison tests using Prism software.

#### Quantification of the GFP^+^ Axon Misprojection Phenotype

**FIGURE 3:**
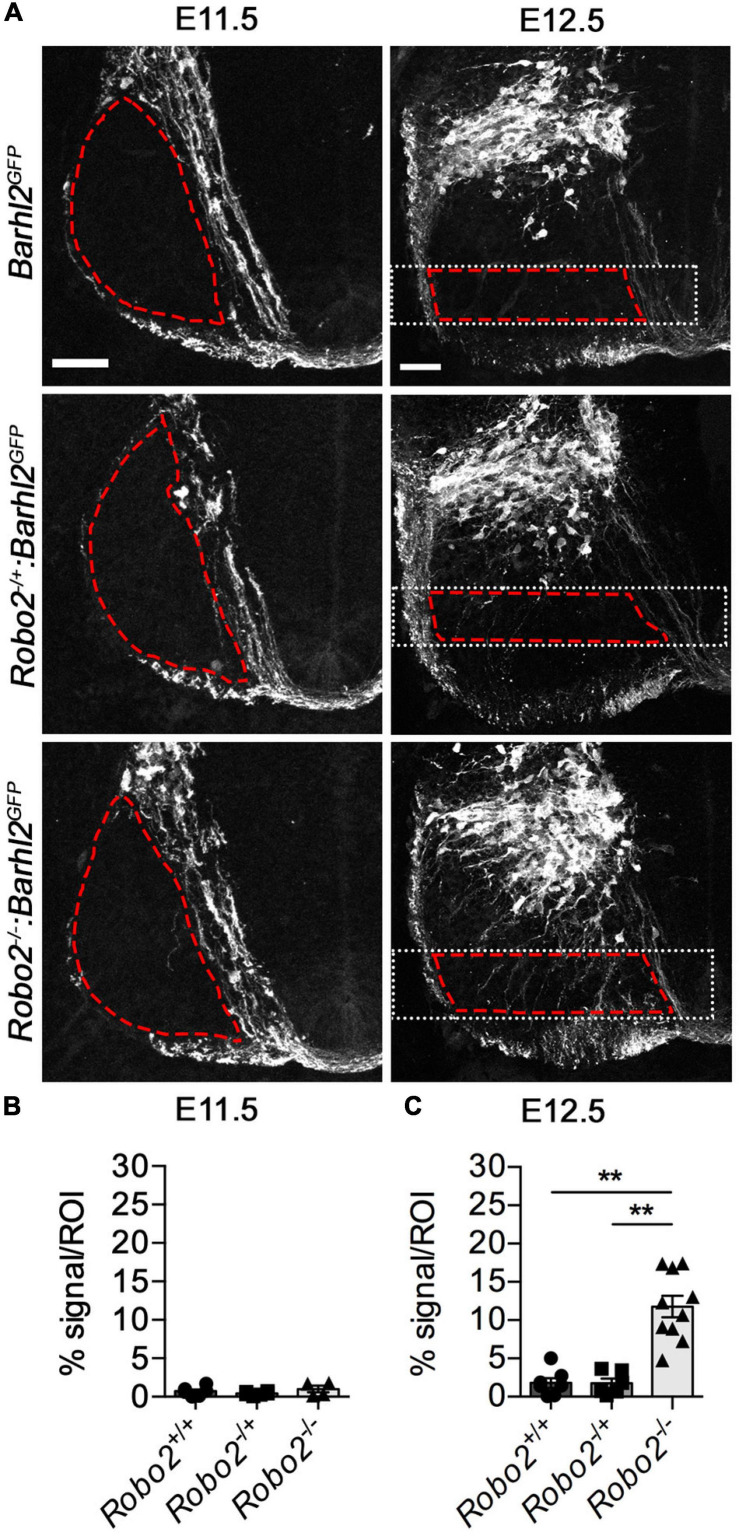
Quantification of dI1 axonal misprojections within the mantle zone of *Robo2^–/–^:Barhl2^*GFP*^* and control embryos. **(A)** Example photomicrographs of mouse E11.5 (left panels) and E12.5 (right panels) spinal cord tissue immunohistochemically labeled with GFP (expressed in dI1 neurons, white) in control *Barhl2^*GFP*^* (E11.5 *n* = 6, E12. 5 *n* = 7), *heterozygote Robo2^+/–^:Barhl2^*GFP*^* (E11.5 *n* = 6, E12. 5 *n* = 6), and *mutant Robo2^–/–^:Barhl2^*GFP*^* (E11.5 *n* = 4, E12. 5 *n* = 10) embryos. The area demarked by the red dashed lines represents the area quantified region of interest (ROI). The white dotted boxed area in the E12.5 images depict the dorsal and ventral boundaries used for quantification (described in the methods). **(B,C)** Quantification of the GFP^+^ axons misprojecting into the ROI is expressed as pixel density per area is shown. This was measured for all genotypic groups, at brachial level for E11.5 and E12.5 in control *Barhl2^*GFP*^* (black circle, E11.5 *n* = 6, E12.5 *n* = 7 embryos), *heterozygote Robo2^+/–^:Barhl2^*GFP*^* (black square, E11.5 *n* = 6, E12.5 *n* = 6 embryos), and *mutant Robo2^–/–^:Barhl2^*GFP*^* (black triangle, E11.5 *n* = 4, E12.5 *n* = 10 embryos). This measurement is referred to on the graph as % signal per ROI. Mean, standard errors, and statistical significance of Kruskal–Wallis test are shown. ** represent a *p* < 0.01. Scale bars in **(A)** are 50 μm and represents the images within the same embryonic ages, E11.5 and E12.5, respectively.

Photomicrographs of E11.5 were imaged. The region of interest was defined by delimiting the white mater tracts as the outer border and commissural axons tract as the inner boundry respectively. Z-stack photomicrographs (20×) of E12.5 mouse embryonic spinal cord tissue immunohistochemically labeled with GFP (dI1 neurons) were acquired at subthreshold pixel intensity with respect to GFP^+^ axons using a Zeiss LSM 710 confocal microscope. From these images, using an ImageJ macro (available on request), the pixel density was measured within a defined region where dI1 axons and cell bodies were normally absent or observed at a low frequency in control embryos. In short, the area to be analyzed was first defined to exclude cell bodies on all sections analyzed. First, a rectangular region was defined which was delimited by the midline on the medial side and by the bottom of the floor plate on the ventral side (white dotted rectangles in Figure 3). The same size and positioning of the rectangle were used for each sample. Within the selected rectangular area, the regions of interest (ROI) to be analyzed were selected. The ROI were selected by manually drawing an area (red dashed lines) within the rectangles’ ventral and dorsal edges, commissural projections on the medial side, and the border of the gray matter adjacent to the white matter tract on the lateral side as borders (dashed red lines in Figure 3). By this method, only the gray matter and not the white matter was analyzed. The ROI were then binarized using the same threshold for all samples, and the background was standardized using the median filter to 1 pixel using a standard macro for all samples (available on request). Each value was then divided by the ROI area to generate a value per area unit. Embryos of the following genotypes were analyzed: *Robo2^+/+^:Barhl2^*GFP*^* (control, *n* = 7), *Robo2^+/–^:Barhl2^*GFP*^* (*n* = 6), *Robo2^–/–^:Barhl2^*GFP*^* (mutant, *n* = 10), *Robo1^+/+^:Robo2^+/+^:Barhl2^*GFP*^* (control, *n* = 8), *Robo1^–/+^:Robo2^–/+^:Barhl2^*GFP*^* (heterozygous, *n* = 8), and *Robo1^–/–^:Robo2^–/–^:Barhl2^*GFP*^* (mutant, *n* = 8) embryos. Values from a single embryo were averaged to generate a value for that embryo. The “n values” refer to the number of embryos analyzed for each genotypic group. Values for each embryo analyzed were averaged (mean) for all embryos of a single genotype. The statistical analysis was performed using GraphPad (Prism) software. The data distribution was non-parametric, and the following statistical test was performed to examine statistical differences: Kruskal–Wallis test followed by Dunn’s multiple-comparison test using Prism software.

**FIGURE 4:**
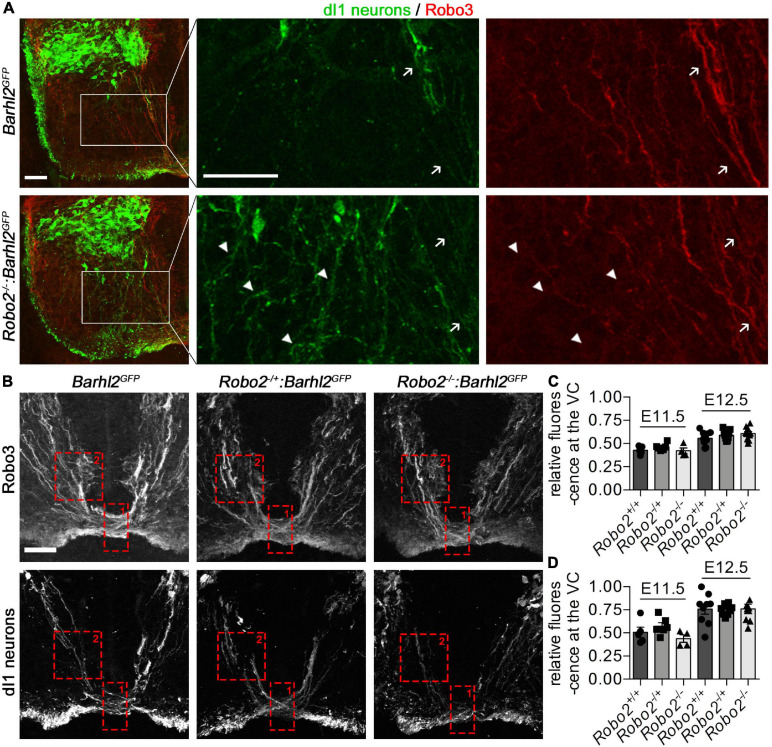
Misprojecting GFP^+^ dI1i axons are Robo3 negative in *Robo2^–/–^:Barhl2^*GFP*^* embryos. **(A)** Photomicrographs of control *Barhl2^*GFP*^* (*n* = 6) and *mutant Robo2^–/–^:Barhl2^*GFP*^* (*n* = 10) embryonic brachial level spinal cord tissue (E12.5) immunohistochemically labeled with Robo3 (expressed in commissural neurons, red) and GFP (expressed in dI1c and dI1i neurons, green). The boxed areas in the left panels are enlarged in the single-channel images. White arrows point to GFP^+^/Robo3^+^ dI1c neurons. White arrow heads point to GFP^+^/Robo3*^–^* misprojecting dI1i neurons. Representative images are shown. **(B)** Photomicrographs of E12.5 brachial-level ventral commissure spinal cord tissue immunohistochemically labeled with Robo3 (expressed in all spinal commissural neurons, upper panels) and GFP (expressed in dI1 neurons, lower panels) in control (*Barhl2^*GFP*^*, *n* = 9), Robo2 heterozygote (*Robo2^+/–^:Barhl2^*GFP*^*, *n* = 9), and *Robo2* mutant (*Robo2^–/–^:Barhl2^*GFP*^*, *n* = 10) embryos. **(C,D)** Quantification of commissural axons. The regions quantified are depicted with a red dotted box for ventral commissure (Box 1) and pre-crossing commissural axons (Box 2). The values quantified are for all Robo3^+^ commissural axons **(C)** and GFP^+^ dI1c axons **(D)** crossing the ventral midline expressed as a ratio of precrossing to crossing pixel intensity which was measured for all genotypic groups, at E11.5 and E12.5 in control *Barhl2^*GFP*^* (black circle, E11.5 *n* = 5, E12.5 *n* = 9 embryos), *heterozygote Robo2^+/–^:Barhl2^*GFP*^* (black square, E11.5 *n* = 6, E12.5 *n* = 9 embryos), and *mutant Robo2^–/–^:Barhl2^*GFP*^* (black triangle, E11.5 *n* = 5, E12.5 *n* = 10 embryos) brachial-level ventral commissure (VC) spinal cord tissue. Mean and standard errors are shown. Scale bars in **(A,B)** are 50 μm. The small bar in **(A)** corresponds to the low-magnification images (first panel for each genotype), and the large scale bar corresponds to the enlarged single-channel images. The scale bar in **(B)** corresponds to all images in **(B)**.

## Results

### Robo2 Expression is Enriched in dI1 Ipsilateral vs. dI1 Commissural Neurons

In order to determine if the expression of Robo receptors was a mechanism gating divergence of neurons from a common precursor origin, we first examined Robo expression during dI1 neuron development (Figures 1A,B). To this end, we generated and validated antibodies against Robo1, Robo2, and Robo3 and analyzed their expression in the spinal cord of either *wild type* or *Barhl2^*GFP*^* mouse embryos, a previously characterized transgenic mouse line which expresses GFP in both dI1 neuron axons and cell bodies (Figures 1C–E and Supplementary Figures 1–4). As previously shown, in E11.5 pre-crossing dI1c and other spinal commissural axons within the gray matter, Robo1 and Robo2 were weakly expressed whereas Robo3 was strongly expressed (Supplementary Figures 1E,F, 2C; Long et al., 2004; Sabatier et al., 2004; Johnson et al., 2019). By E12.5, a key choice point in the anatomical divergence of dI1c and dI1i neurons Robo2 was observed to be expressed in a stereotyped manner within the deep dorsal horn in a pattern intriguingly reminiscent of the position of dI1i neurons (Supplementary Figure 3). This was in sharp contrast to Robo3 which has previously been shown to be exclusively expressed in commissural neurons (Supplementary Figure 3; Marillat et al., 2004; Sabatier et al., 2004). Strikingly, using *Barhl2^*GFP*^* embryos to track dI1c and dI1i neurons revealed that Robo2 was indeed expressed in GFP^+^ dI1 neurons within the mantle zone of the spinal cord (Figure 1D). In particular, Robo2 was enriched in dI1i neurons and more weakly expressed in dI1c neurons (Figures 1D,I,J). In sharp contrast, Robo3 protein and mRNA were not detected in dI1i neurons whereas Robo1 was expressed in both dI1c and dI1i neurons (Figures 1C,E,F,H,J and Supplementary Figure 3). Taken together, these data revealed that the expression profiles of Robo2 and Robo3 receptors bifurcate at a key choice point in the divergence of ipsilateral (Robo2^+^/Robo3*^–^*) vs. commissural (Robo2^*l*ow^/Robo3^+^) dI1 neurons whereas Robo1 was expressed in both dI1i and di1c neurons with a modestly higher expression dI1c compared with dI1i neurons (Figure 1J). This suggested a potential complementing role of canonical Robo2 vs. non-canonical Robo3 receptors in dI1c and dI1i response to the environment and anatomical divergence.

### dI1 Cell Bodies Migrate Aberrantly in *Robo2* Mutant Embryos

Given the enrichment of Robo2 in dI1i neurons, we next explored the possibility whether the canonical Robo2 receptor influenced dI1 guidance. For this analysis, we crossed the *Barhl2^*GFP*^* transgene into *Robo2*^+/–^ mice. The embryos from these crosses are referred to here as *Robo2* mutant (*Robo2^–/–^:Barhl2^*GFP*^*), *Robo2* heterozygote (*Robo2^+/–^:Barhl2^*GFP*^*), and control (*Barhl2^*GFP*^*) embryos.

To determine if Robo2 influenced dI1 neuronal migration, we first immunohistochemically labeled *Robo2* mutant and control embryos with a transcription factor delineating dI1 neuron nuclei, Barhl2 (pseudocolored in green in Figure 2; Ding et al., 2012). Barhl2 is expressed in dI1 neurons as defined by expression of Lhx2 and Lhx9 transcription factors and lack of expression of markers of dI12–dI16 neurons (Ding et al., 2012). At the ages used here, Barhl2 is a preferable marker for the analysis to Lhx2 or Lhx9 since, unlike Lhx2 and Lhx9, Barhl2 labels dI1 neurons more broadly. At E11.5, a time at which dI1 neuron ventral cell body migration is underway but before they have settled in the deep dorsal horn or diverged into dI1c and dI1i populations, we did not observe a dI1 neuron migration phenotype in *Robo2* mutant embryos (Figure 2A). In sharp contrast, at E12.5 dI1 neuron migration was profoundly disrupted in *Robo2* mutant embryos (Figure 2B). In E12.5 control embryos, cell bodies of dI1 neurons had stereotyped positions within the deep dorsal horn, with a segregation of medial, dI1c neurons and lateral, dI1i neurons, whereas in *Robo2* mutant embryos many Barhl2^+^ dI1 cell bodies were misplaced in the ventral horn and/or accumulated in a more medial position (Figure 2B). To quantify this phenotype, images of E12.5 brachial sections were analyzed from different genotypic groups and the relative distribution of Barhl2 antibody labeling was determined (Figure 2C). The total number of Barhl2^+^ cells was not statistically significantly different between genotypes, indicating that shifts in cell distribution were due to migration and not a changed cell number (Supplementary Figure 5). While the individual data points for each individual bin/genotype are shown in Supplementary Table 1, this quantitative analysis was most effectively summarized in a heat map form showing the distribution of Barhl2^+^ neurons in control and *Robo2* mutant embryos (indicated by green color intensity in Figure 2C and Supplementary Table 1). The mean number of Barhl2^+^ neurons per bin is indicated in Figure 2C; bold values indicate a statistically significant difference between control and mutant embryos and the direction of cell number change indicated by an up or down arrow for decreased or increased number of nuclei, respectively. This analysis, consistent with the qualitative observations, revealed a pronounced and statistically significant medial/ventral distribution shift of Barhl2^+^ neurons in *Robo2* mutant compared to control embryos (Figure 2C and Supplementary Table 1). Interestingly, labeling of E12.5 Robo2 mutant and control samples with the transcription factor Lhx2 which at the age and axial level analyzed is restricted to dI1c neurons did not show differences in the position of dI1c cell bodies (Supplementary Figures 6, 7A). In contrast, labeling with Lhx9 revealed a ventral shift in Lhx9^+^ neuron cell body position (Supplementary Figures 6, 7A,B; Wilson et al., 2008). These data were consistent with the notion that Robo2 was selectively influencing dIli but not dI1c migration.

The above data supported the notion that canonical Robo2 signaling in dI1 neurons influenced their guidance. This suggested a mechanism whereby an inhibitory boundary prevents dI1 neurons from entering the ventral horn. Classically repellent canonical Robo2 signaling is activated by Slit ligands. Of the three mammalian Slit ligands, *Slit2* is known to be expressed in a region ventral to developing dI1i neurons as they project ipsilaterally at E12.5 (Supplementary Figure 8; Kadison et al., 2006). This was consistent with the idea that Slit2 may be the ligand responsible for restricting projecting dI1 neurons, preventing them from entering the ventral horn. If this was the case, knocking out the *Slit2* gene would result in migration errors in dI1 neurons similar to that observed for *Robo2* mutant embryos. To address this, we analyzed *Slit2* mutant (*Slit2^–/–^:Math1^*LacZ*^*) and control (*Math1^*LacZ*^*) embryos to determine whether a similar phenotype was observed between *Slit2* and *Robo2* mutant embryos. Equivalent to the analysis for the *Robo2* mutant embryos, we analyzed the distribution of Barhl2 nuclei in *Slit2* mutant embryos. We found a notable medial shift in the population distribution on *Slit2* mutant embryos, consistent with a medial shift in the dI1i neural population (Supplementary Figure 9 and Supplementary Table 2). Of note, the effect in *Slit2* mutant embryos was less pronounced than the migration phenotype observed in the *Robo2* mutant embryos, and further we observed a statistically significant medial but not ventral shift in the Barhl2^+^ cell population. Moreover, unlike the *Robo2* mutant analysis where several ventral regions of significance were observed in the *Slit2* mutant embryos, fewer bins showed a statistically significant increase or decrease in Barhl2^+^ neurons and statistically significantly less strong (Supplementary Figure 9 and Supplementary Table 2). Nevertheless, the reduction in Barhl2^+^ dI1 neurons in the lateral spinal cord, a position normally occupied exclusively by dI1i neurons suggested that dI1i neurons were mismigrating in both *Robo2* and *Slit2* mutant embryos. Overall, this suggested that Robo2 was controlling dI1 neuronal migration and that the canonical Slit2 was a ligand contributing in part of Robo2’s influence over dI1 neuronal migration.

### Axon Guidance Errors of dI1 Neurons in *Robo2* Mutant Embryos

Next, to determine if Robo2 influenced dI1 axon guidance within the mantle zone, we delineated dI1c and dI1i axons with GFP derived from the *Barhl2^*GFP*^* transgene. At E11.5, a time at which dI1c neurons have started to project axons across the floor plate but before dI1i neurons have projected ipsilaterally, no GFP^+^ dI1 neuron misprojection phenotypes within the ventral horn were observed in *Robo2* mutant compared with control embryos (Figure 3A). In sharp contrast by E12.5, the morphology of dI1 GFP^+^ axons was strikingly different between *Robo2* mutant and control embryos (Figure 3A). We observed that GFP^+^ dI1 axons appeared to project axons indiscriminately through the ventral horn in a disorganized manner in *Robo2* mutant embryos compared with control embryos (Figure 3A). In some cases, unlike control dI1 neurons, which were generally straight in appearance, misprojecting axons in *Robo2* mutant embryos projected with a meandering and crooked appearance. To analytically quantify the observed axonal phenotype further, we imaged dI1 GFP^+^ neurons in*Robo2* mutant, *Robo2* heterozygote, and control embryos at equivalent axial levels (brachial levels at E11.5 and E12.5) and measured pixel density in defined regions (area delineated by red dashed lines in Figures 3A–C). At E11.5, no difference in GFP^+^ axons in the ventral horn was observed regardless of genotype (Figure 3B). In stark contrast, at E12.5, a statistically significant increase of GFP^+^ axons entering the ventral horn was measured in *Robo2* mutant compared with heterozygote or control embryos (Figure 3C). These data confirmed the above qualitative observations that dI1 axons were robustly misplaced in *Robo2* mutant embryos.

For the analysis with *Slit2* mutant embryos, the *Math1^*LacZ*^* transgene was used as a dI1 marker instead of the *Barhl2^*GFP*^* transgene used in *Robo2* mutant embryos, since the *Slit2* mouse line has GFP knocked into the gene locus rendering the use of a GFP transgene inappropriate. The *Math1^*LacZ*^* and *Barhl2^*GFP*^* transgenes both label dI1 neurons (Supplementary Figure 4; Helms and Johnson, 1998; Wilson et al., 2008). While E12.5 dI1 cell bodies are clearly visible using *Math1^*LacZ*^*, unlike GFP in the *Barhl2^*GFP*^* transgene, the ß-galactosidase expression in the dI1i axons in *Math1^*LacZ*^* E12.5 embryos was too weak for the potential dIi misprojecting axons to be analyzed. Therefore, here we focused on the migration analysis.

Taken together, this provided evidence that Robo2 influenced dI1 neuron guidance. Importantly, absence of dI1 misprojecting axons within the ventral horn of E11.5 *Robo2* mutant embryos revealed that Robo2’s action was at a specific choice point in dI1 development a time at which dI1i neurons are undergoing mediolateral axonal projection and the cell body positioning of dI1i and dI1c cell body starts to diverge.

### dI1 Ipsilateral Neurons are Misguided in *Robo2* Mutant Embryos

The GFP in the *Barhl2^*GFP*^* transgene used in this study labels both ipsilateral and commissural projecting developing dI1 neurons, meaning that the misprojecting axons observed could be dI1c or dI1i neurons. We found that canonical Robo2 was enriched in dI1i neurons and that in the *Robo2* mutant embryos the ectopic axons start to appear at a developmental time point as dI1i neurons begin to anatomically diverge from dI1c neurons (E12.5), supporting the notion that the phenotypes were derived from the ipsilateral population. Further, we observed that in the *Robo2* mutant embryos, commissural axon projections were projecting relatively normally within the mantel zone at E11.5 and E12.5, suggesting that dIc neurons within the mantle zone remained unperturbed (Figure 4). However, of note, previous work has shown that canonical Robo receptors modulate commissural axon projections in mice, whose subtype of commissural neurons is not known (Long et al., 2004; Jaworski et al., 2010; Johnson et al., 2019). This knowledge, taken together with our finding that Robo2 was detected in dI1c neurons, albeit at a lower level than in dI1i neurons, raised the possibility that the observed misprojection phenotypes in the *Robo2* mutant embryos could be commissural or ipsilateral specific, or an alternative possibility was that the phenotype was caused by misguidance of both populations (Figures 1, 2). To distinguish between these possibilities, we labeled *Robo2* mutant and control embryos with a commissural neuron-specific antibody, Robo3 (red in Figure 4), together with GFP to delineate dI1 neurons (green in Figure 4). We observed that the projection of GFP^+^/Robo3^+^ dI1c neurons appeared similar between *Robo2* mutant and control embryos (Figure 4A). However, we did not observe obvious dI1 commissural axon phenotypes at a gross level. Since previous work has shown that Robo2 contributes to commissural axon trajectory in coordination with Robo1, prompted us to next analyze this aspect further. To do this, we measured both Robo3^+^ (all commissural neurons) and GFP^+^ (dI1 neurons) immunolabeled axons within the mantle zone and ventral commissure using pixel intensity as a proxy for axon density. The ratio of the pre-crossing/crossing (Box2 vs. Box1 in Figure 4B) GFP^+^ or Robo3^+^ axons was then compared between *Robo2* mutant and control embryos (Figures 4C,D). At E11.5 and E12.5, no significant difference in either GFP or Robo3 pixel intensity ratio in the ventral commissure of the *Robo2* mutant compared with control embryos was measured (Figures 4C,D). Taken together, this suggested that dI1c-crossing axons were not significantly affected in *Robo2* mutant embryos.

**FIGURE 5:**
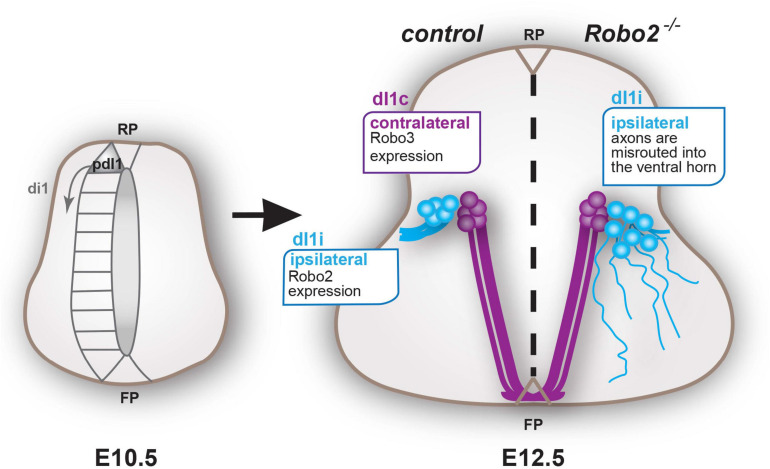
Model schematic summary: dI1 neurons are generated from the pdI1 precursor domain from E10.5 onward. By E12.5, non-canonical Robo3 vs. canonical Robo2 becomes differentially enriched in dI1c (purple) and dI1i (blue) neurons, respectively, permitting dI1c and dI1i neurons to respond uniquely to their environment and thus diverge anatomically. Abbreviations are as follows; dorsal interneuron 1, precursors, commissural and ipsilateral neurons (pdI1, dI1c, and dI1i, respectively), roof plate (RP), and floor plate (FP).

In stark contrast to GFP^+^/Robo3^+^ dI1c neurons, which appeared to project normally in *Robo2* mutant compared with control embryos, GFP^+^/Robo3*^–^* dI1i neurons misprojected into the ventral horn (Figure 4A, white arrowheads). Taken together, these observations suggested that the misprojection phenotypes described were ipsilateral specific (Figure 5).

### Robo1 and Robo2 are Not Redundant in Determining dI1 Guidance

In addition to Robo2, our analysis revealed that Robo1 was expressed in dI1 neurons, which raised the possibility that Robo1 could also play a role in dI1 neuron guidance (Figures 1, 2 and Supplementary Figure 3). To examine this possibility, we asked if the absence of *Robo1* enhanced the phenotypes observed in *Robo2* mutant embryos in *Robo1/2* double mutant (*Robo1^–/–^:Robo2^–/–^:Barhl2^*GFP*^*), *Robo1/2* double heterozygote (*Robo1^+/–^:Robo2^+/–^:Barhl2^*GFP*^*), and control (*Barhl2^*GFP*^*) embryos. Using comparable analysis to that of *Robo2* mutant embryos, we observed both axon guidance and dI1 neuron migration misguidance phenotypes in *Robo1/2* double mutant compared with control embryos (Supplementary Figures 10, 11).

Similar to the analysis for *Robo2* mutant embryos, we observed a statistically significant increase of GFP^+^ axons entering the ventral horn in *Robo1/2* double mutant compared with control embryos (Supplementary Figures 10A,B). While, there was a modest increase in the quantified mean value of misprojecting GFP^+^ axons within the ventral horn in *Robo1/2* double mutant embryos compared with the equivalent value in *Robo2* mutant embryos; this was not statistically significant (Supplementary Figure 10C). Overall, these data suggested that the dI1i axon misprojection phenotype observed in the *Robo1/2* double mutant embryos was predominantly driven from loss of the *Robo2* gene (Supplementary Figure 10).

Analysis of the distribution of Barhl2^+^ cell bodies in *Robo1/2* double mutant and control embryos revealed that the dI1 migration phenotype by loss of both *Robo1* and *Robo2* was more pronounced than loss of *Robo2* alone (Figure 2, Supplementary Figure 11, and Supplementary Tables 1, 3). In particular, Barhl2^+^ neurons migrated more ventrally in *Robo1/2* double mutant compared with *Robo2* mutant embryos (Supplementary Figure 11 and Supplementary Table 3). Of particular note, we observed that there was a statistically significant migration defect in *Robo1/2* double heterozygote vs. control embryos (Supplementary Table 3). However, this effect was much less pronounced compared with *Robo2* mutant or *Robo1/2* double mutant embryos, suggesting that this double heterozygote migration phenotype was not simply due to a gene-dose effect. Taken together, this suggested that Robo1 and Robo2 did not have redundant roles in this context and that Robo2 was the major contributor to dI1i axon guidance.

## Discussion

The focus of this study was to determine the mechanisms underlying how projection neurons derived from a common precursor origin read out their environment uniquely to elaborate correct spatial organization and connectivity. Using developing spinal dI1 projection neurons as a model system, we tested the hypothesis that the choreographed expression of Robo receptors served as a mechanism to drive anatomical divergence of neurons from a common precursor origin (Figure 5).

### Robo2 Selectively Gates dI1i Guidance Within the Mantle Zone

The most important finding from this study was the observation that within the mantle zone, dI1i neurons were profoundly misguided in *Robo2* mutant embryos, whereas dI1c neurons appeared to project relatively normally toward and within the ventral commissure. The conclusion that the phenotype observed was ipsilateral specific was reached through various lines of evidence and reasoning. dI1i neurons are born in the dorsal spinal cord from the pdI1 domain and initially migrate ventrally before migrating laterally and projecting axons ipsilaterally at E12.5 (Figures 1A,B, 5; Wilson et al., 2008). Favoring the idea that the misprojection phenotypes observed were subtype specific, we noted that in *Robo2* mutant embryos the emergence of dI1-misprojecting axons and mislocalized cell bodies was at E12.5, indicating that the phenotype onset was coincident with the emergence of ipsilateral projections and lateral migration of cell bodies of the dI1i population but after dI1c neurons emerged. The medial shift in the positioning of dI1 neurons in *Robo2* and *Slit2* mutant embryos meant that the number of dI1 neurons occupying the lateral position normally populated by dI1i neurons was significantly reduced, suggesting that dI1i neural migration was influenced by the Robo2/Slit2 axis. It has previously been shown that Robo signaling influences precursor cell dynamics within the ventricular zone (Borrell et al., 2012). However, in our analysis of the mantle zone we did not observe statistically significant differences in dI1 neuron number regardless of genotype, suggesting that the primary phenotype observed was due to a migration defect in dI1 cell body position and not a change in overall neuron number. Importantly, the *Robo2* mutant transgenically labeled misprojecting axons were negative for the commissural axon-specific marker Robo3, consolidating the view that these misprojecting axons were indeed from the dI1i neuron population. The observation that Robo2 was substantially enriched in dI1i vs. dI1c neurons supported the notion that Robo2 was indeed influencing dI1i neurons.

In contrast to dI1i neurons within the mantle zone, we did not observe dI1c misprojection phenotypes in the gray matter or floor plate of *Robo2* mutant embryos. In particular, we noted that in E11.5 embryos, at a time when dI1c neurons are projecting across the midline but before dI1i ipsilateral projections were observed, the relative proportion of transgenically labeled GFP or Robo3 axons within the ventral commissure was similar regardless of genotype, indicating that dI1c projections were grossly normal (Figure 4). A similar finding was observed at E12.5 (Figure 4). Previous studies have shown that in mice, *Robo1* knockout embryos have a mild spinal commissural axon-recrossing phenotype whereas *Robo2* mutants cross normally, and instead Robo2 has been proposed to regulate post-crossing commissural axon projections (Jaworski et al., 2010). Consolidating this assertion, it has also been demonstrated that, in mice, unlike *Robo1*, modulation of Robo2 (either loss or gain of function) does not produce a mantle zone or ventral commissure commissural axon phenotype unless Robo1 is modulated at the same time (Long et al., 2004; Jaworski et al., 2010; Johnson et al., 2019). In this way, Robo1 appears to play a more leading role in spinal commissural guidance within the gray matter and ventral commissure whereas Robo2 acts to synergize with Robo1 possibly through Robo1^+^ pioneer axons guiding Robo2^+^ follower axons. Indeed, which populations of commissural neurons express Robo1 and Robo2 has not been well described. Here we provide evidence that dI1c neurons express Robo1 and a low level of Robo2. However, we did not observe a dI1c or other commissural neuron axonal projection phenotype in the mantle zone or ventral commissure in *Robo2* mutant embryos (Figures 1, 4, and Supplementary Figure 1). Finally, we did not observe differences in the position of Lhx2-expressing cells, which at the age and level examined has previously been shown to label dI1c but not dI1i neurons between *Robo2* mutant and control embryos, whereas Lhx9^+^ neurons were mislocalized (Wilson et al., 2008). This suggested that dI1c neurons’ position was unperturbed in *Robo2* mutant embryos and is consistent with the idea that dI1i neural cell bodies were mislocalized. Taken together, these data supported the notion that within the mantle zone Robo2 was selectively influencing dI1i neurons whereas dI1c neurons remained unperturbed.

### Canonical Robo2 vs. Non-Canonical Robo3 Receptors Influence dI1i vs. dI1c Respectively to Elicit Neural Organization

The results in this study taken together with previous work suggest that canonical vs. non-canonical Robo signaling coordinate to sculpt dI1 neuron organization. One study in *Drosophila melanogaster* has suggested a non-canonical role for Robo2 in commissural axon crossing whereas in mammals Robo2 has been shown to function as a canonical Robo receptor (Evans et al., 2015). However, it should be noted that the *Robo2* genes in *Drosophila melanogaster* and vertebrates have different evolutionary generations (Friocourt and Chedotal, 2017). In respect to canonical Robo function, we observed that a canonical Robo ligand, Slit2, was expressed in a region ventral to dI1i neurons, and further, dI1 neurons migrated aberrantly in *Slit2* mutant embryos. Of note, the *Slit2* migration phenotype was mild compared with the *Robo2* mutant embryos. Barhl2^+^ neurons had a medial but not ventral shift in *Slit2* mutant embryos, unlike the *Robo2* mutant embryos which had both a medial and ventral shift in this population. A speculation could be that Slit2 diffusing dorsally from the medially located floor plate might account for this medial shift. The data suggests that the medial and ventral guidance of dI1 neurons by Robo2 could be directed by different ligands. The data suggested that Slit2 may be redundant with other Slit ligands such as Slit1 and Slit3, which are both expressed at the mRNA level within the ventral spinal cord mantle zone at earlier time points. Another Robo ligand, Nell2, has been found to be present within the ventral horn at E11.5 and E12.5 (Jaworski et al., 2015). However, Nell2 has been shown to bind to the non-canonical Robo3 receptor by virtue of evolution of the fibronectin III domain of Robo3, thus providing a chemo barrier to entry of Robo3^+^ commissural axons into the ventral horn (Jaworski et al., 2015; Pak et al., 2020). While Nell2 has been shown to influence Robo3 signaling, given that a biochemical study has demonstrated that under specific conditions, Robo2, but not Robo1, can bind to Nell2, leaving open the possibility that Nell2 acts as a functional ligand for Robo2, although to date that has not been determined (Yamamoto et al., 2019). Taken together, this indicated that Slit2 is a contributing ligand regulating Robo2-mediated dI1 guidance and is likely that either the ligand(s) above or yet unidentified ones are also playing a role.

Fundamental for the overall mechanism, previous work has shown that spinal commissural axons (including dI1c) within the mantel zone are gated by the expression and activity of the non-canonical Robo3.1 receptor (Sabatier et al., 2004). This, taken together with the work presented here, provides a mechanism whereby canonical Robo2 and non-canonical Robo3 are differentially expressed at a key anatomical divergence point resulting in dI1i and dI1c neurons’ ability to respond uniquely to their environment (Figure 5).

### Robo Signaling in the White Matter Later in Development

Here we examine how neurons derived from a common precursor origin respond uniquely to the environment utilizing Robo signaling. A cohort of previous work has demonstrated that embryonic axonal sorting in their longitudinal white matter tracts is influenced by Robo1 and Robo2 (Long et al., 2004; Devine and Key, 2008; Reeber et al., 2008; Jaworski et al., 2010; Mastick et al., 2010; Kim et al., 2011; Sakai et al., 2012). These studies focused on the longitudinal white matter tracts, which refers to a later developmental choice point for the axons compared with the analysis in this paper. Further, unlike the study presented here, these former studies do not address how neurons derived from a common population respond uniquely to their environment. An elegant study in the mouse hindbrain has found that pontine neurons in the gray matter migrate in a cell non-autonomous manner (Dominici et al., 2018). However, unlike the study by Dominici et al., we observed that Robo2 was expressed in the mantle zone in the neurons that misprojected in *Robo2* mutant embryos’ dI1i neurons. This suggested that within dI1 neurons, the evidence points to the phenotype observed in the Robo2 mutant embryos within the spinal cord as a cell autonomous effect.

The expression of Robo2 (and Robo1) we observed in dI1 neurons within the mantle zone was substantially weaker than that observed in the latter projecting white matter tracts (Figure 1; Long et al., 2004). This most likely reflects both the tight regulation of Robo receptors and the potency of Robo signaling within the mantle zone. Within the white matter tracts, it has been shown that Robo1 expression is regulated at the translational level and translational regulation of Robo2 has also been inferred (Long et al., 2004; Yang et al., 2018). Our data showed that *Robo1* and *Robo2* mRNA and protein broadly correlated in dI1i neurons within the mantle zone, suggesting that in contrast to white matter tracts, the regulatory mechanism within the mantle zone may be at the transcriptional level. Several transcription factors are candidates that could potentially regulate this; however, this is likely to be complex transcriptional regulation and has not been determined (Wilson et al., 2008; Ding et al., 2012; Marcos-Mondejar et al., 2012; Escalante et al., 2013).

### Robo2 is the Major Player in dI1i Guidance Within the Mantle Zone

We demonstrated that in addition to Robo2, Robo1 was expressed in both dI1i and dI1c neurons, raising the possibility that Robo1 influenced dI1 guidance. We found that loss of *Robo1* did not statistically significantly enhance the *Robo2* mutant dI1i axonal phenotype, suggesting that Robo2 alone was sufficient for the observed axonal phenotype. Of note, while not statistically significant, we observed a marginal mean increase (trend) in axonal misprojections in *Robo1/2* double mutants compared with *Robo2* mutant embryos. This evidence leaves open the possibility that Robo1 may contribute to the projection of dI1i neurons, which could be for example by contributing to a “threshold” of canonical Robo signaling. We also noted a qualitative but not quantitative dI1i axonal phenotype in *Robo1/2* double-heterozygote embryos compared with controls. This modest axonal phenotype *Robo1/2* double-heterozygote embryos (two Robo gene copies missing) compared with the strong phenotype in Robo2 mutant embryos (two Robo gene copies missing) suggested that the influence of Robo1 and Robo2 were not equal in dI1i neurons. This suggested that while canonical gene dose may contribute to the guidance, canonical Robos’ influence on dI1i guidance is not mediated by gene dose alone. Taken together, we therefore concluded that Robo2 and Robo1 are not redundant in the context of dI1i axonal projections within the mantle zone and that Robo2 plays the major role. We found a statistically significantly enhanced cell body migration phenotype in E12.5 *Robo1/2* double mutant vs. *Robo2* mutant embryos, suggesting that in the context of the cell body migration phenotype, Robo1 and Robo2 may be additive. Consistent with our analysis overall, in other contexts, Robo1 and Robo2 can act in a distinct, synergistic, or redundant way depending on the context (Hivert et al., 2002; Gruner et al., 2019).

This, taken together with other studies, suggests that Robo1 may play a role in commissural axon timing and crossing at the ventral commissure, and here we propose that Robo2 plays a relatively minor role in dI1c neuron guidance within the mantle zone and ventral commissure whereas Robo2 is the major gatekeeper of dI1i guidance.

## Concluding Remarks

Robo signaling is associated in a wide range of fields including neurodevelopment and neurofunctional disorders in addition to disease processes. Here we have contributed to this body of work demonstrating a neurodevelopmental context in which canonical vs. non-canonical Robos regulate tissue organization. Within the nervous system, understanding how neurons derived from common precursor origin elaborate neural organization has important implications for understanding the assembly of functional connectivity. This study provides an example of how evolution of a gene family permits development of anatomical differentiation/identity from cells derived from a common progenitor pool and initial molecular identity, a concept that is widely applicable throughout different systems and animals. There are a small number of examples of the non-canonical function of Robo receptors in other species (Burgess et al., 2009; Evans et al., 2015). Whether the model we have described of canonical vs. non-canonical Robo function has a similar role in non-mammalian species remains to be determined.

## Data Availability Statement

The original contributions presented in the study are included in the article/Supplementary Files, further inquiries can be directed to the corresponding author.

## Ethics Statement

The animal study was reviewed and approved by Animal Review Board at the Court of Appeal of Northern Norrland.

## Author Contributions

MW, MM, LG-C, and CL assembled the figures. MW, MM, and LG-C were responsible for writing parts of the manuscript, experimental design, and performance of experiments. MW performed most of the experiments and analysis in the manuscript and codesigned and performed the phenotype quantifications. MM made the initial *Robo2* phenotype discovery and preliminary analysis and generated reagents. LG-C designed and performed the Robo1, Robo2 and Robo3 expression quantification, contributed to the migration analysis, characterization of the transgenes and antibodies. CL and CT were involved in an early phase of analysis in the work and generated samples. SW conceived and supervised the work, led the study design, generated the funding, made reagents, performed some experimental work and formal analysis, and wrote the manuscript. All the authors edited, read, and approved the manuscript.

## Conflict of Interest

The authors declare that the research was conducted in the absence of any commercial or financial relationships that could be construed as a potential conflict of interest.

## Abbreviations

pdI1: precursor domain dorsal interneuron 1
dI1: dorsal interneuron 1
dI1c: dorsal interneuron 1 commissural neurons
dI1i: dorsal interneuron 1 ipsilateral neurons.
