# Supplementary material for: Robo2 Receptor Gates the Anatomical Divergence of Neurons Derived From a Common Precursor Origin
Source: Front Cell Dev Biol. 2021 Jun 23;9:668175. doi: 10.3389/fcell.2021.668175 (PMC8263054; doi:10.3389/fcell.2021.668175)
Supplement: Supplementary file 1 [file Data_Sheet_1.pdf]

Supplementary Material

1 Supplementary Methods

1.1 Robo antibody production

Antibodies against Robo1 (rabbit), and Robo2 (guinea pig) and Robo3 (rabbit) were produced for this study. Plasmids expressing either rat *Robo1*, *Robo2* or mouse *Robo3* ectodomain tagged with human IgG1 Fc region (EctoRobo1eFc/pCAG3, EctoRobo2eFc/pCAG3 and EctoRig1eFc/pCAG3) were transfected using lipofectamine into HEK293 cells (Robo1 and Robo2) or by DEAE-dextran method into COS-7 cells (**Supplementary Figures S1A - B**, **S3A**) (Tamada et al., 2008). *Robo1* (amino acid 1-892 of Robo1), *Robo2* (amino acid 1- 855 of Robo2) and *Robo3* (amino acid 1- 864 of Robo3) ectodomain DNA constructs were from Professor Fujio Murakami and Dr Atsushi Tamada (Osaka University, Yamadaoka, Japan). Conditioned medium containing Robo ectodomain protein was collected and purified using a Protein A agarose chromatography (Thermo Scientific™ 20333) (**Supplementary Figures S1B**, **S2A**). The Robo1, Robo2 and Robo3 ectodomain/IgG fusion proteins were used to inoculate rabbits (Robo1 and Robo3) or guinea pigs (Robo2) using standard inoculation procedures by Covance, USA (Robo1 and Robo2) or Agrisera AB, Box57, SE-911 21, Vännäs, Sweden (Robo3) (**Supplementary Figures S1, S2**).

1.2 Lhx2 and Lhx9 antibody production

Antibodies against, Lhx2, Lhx9 and were raised in rabbit and Guinea Pig respectively using the following peptides: SLSGPEVHGVIDEMDC (Lhx2) and GISGGHIQGIMEEMEC (Lhx9). Peptides were coupled to Keyhole limpet hemocyanin (Peirce) according to manufactures instructions. All antigens were injected using standard inoculation procedures by Covance, USA (**Supplementary Figures S6**).

1.3 Specificity of the newly produced antibodies

We first validated the specificity of the Robo1, Robo2 and Robo3 antibodies by examining whether the produced antibodies recognized the over-expressed recombinant Robo1, Robo2 and Robo3 in COS-7 cells (**Supplementary Figures S1C, D, S2B**). COS-7 cells were grown, transfected and process as described previously with the following exceptions: Transfection was with either GFP expressing plasmid (*CMV-GFP/pEGFP-N2* and empty vector (*PUC19* or *pCDNA3.1*) or GFP expressing plasmid (*CMV-GFP/pEGFP-N2*) and Robo1, Robo2 or Robo3 ectodomain over-expression plasmids EctoRobo1eFc/pCAG3, EctoRobo2eFc/pCAG3 and EctoRig1eFc/pCAG3 respectively (Kropp and Wilson, 2012). Following transfection and incubation, immunohistochemistry was performed using GFP and Robo1 or GFP and Robo2 or GFP and Robo3 antibodies. Robo1, Robo2 and Robo3 antibodies recognized cells transfected with *Robo1/GFP or Robo2/GFP* or *Robo3/GFP* expression vectors respectively but not cells transfected with a mock plasmid and *GFP* expression vector alone (**Supplementary Figures S1C, D, S2B**). Each condition was performed in technical triplicate. Next, the specificity of the antibodies was examined by performing immunohistochemistry and analyzing the distribution of labeling within a known context, expression in mouse E11.5 embryos (**Supplementary Figures S1E, F, S2C, C'**). We found the expression produced by the antibodies created in this study were consistent with the known

expression pattern of Robo1, 2 and 3 at E11.5. Further, the antibodies produced in this study were directly compared with verified antibodies currently available (**Supplementary Figures S1E, F and S2C**). For this an aliquot of the previously produced Robo1, Robo2 and Robo3 antibodies from Tamada et al 2008, were kindly provided independently from Dr Atsushi Tamada and Professor Marc Tessier-Lavigne (Stanford University, CA, USA). The Robo antibodies generated in this study were compared with previously generated Robo antibodies from Tamada et al 2008 and commercially available Robo2 (R&D Systems AF3147) and Robo3 (R&D Systems AF3076) antibodies. Finally, we observed that the expression of Robo1, Robo2 and Robo3 was similar regardless of whether GFP positive wild type embryonic samples were used consolidating the view that the expression patterns observed were correct (**Figures 1 and Supplementary Figure S3**). Taken together these data validated that the Robo1, Robo2 and Robo3 antibodies produced in this study were specific for the intended targets.

We validated the specificity of the newly produced Lhx2, and Lhx9 antibodies by examining whether each antibody recognized over-expressed protein in COS-7 or HEK293 cells or whether protein was detected in *Lhx2*$^{-/-}$ or *Lhx9*$^{-/-}$ E11.5 embryos. (**Supplementary Figure S6**). To do this *Lhx2* and *Lhx9* plasmids were first generated. In shot for Lhx2, the *Lhx2* insert was obtained by cutting IMAGE clone 6413339 with HincII/EcoRI and the *Lhx9* insert was from the mouse *Lhx9* plasmid used in (Lee et al., 1998) cut with XhoI and EcoR1. The respective inserts were then blunt cloned into the EcoRV site of pMT23 resulting in expression plasmids of Lhx2 and Lhx9 respectively. COS-7 (used for Lhx9) or HEK293 (used for Lhx2) cells were transfected with either empty vector (*pMT23*), empty vector (*pMT23*) with or without GFP expressing plasmid (*CMV-GFP/pEGFP-N2*), Lhx2 expressing plasmid or Lhx9 over-expression plasmids with or without GFP expressing plasmid (*CMV-GFP/pEGFP-N2*). Following transfection and incubation, immunohistochemistry was performed using Lhx2 or Lhx9 antibodies and the nuclei counterstained with DAPI (**Supplementary Figures S6 A - B**). The specificity of the antibodies was also analyzed by immunohistochemistry in *Lhx2* mutant or *Lhx9* mutant E11.5 mouse embryos (**Supplementary Figures S6 C - D**). Taken together this showed that the Lhx2 and Lhx9 antibodies recognized overexpressed protein, reflected the expected expression pattern and not labeling neurons in the respective knockout embryos.

2 Supplementary Figures and Tables

2.1 Supplementary Figures

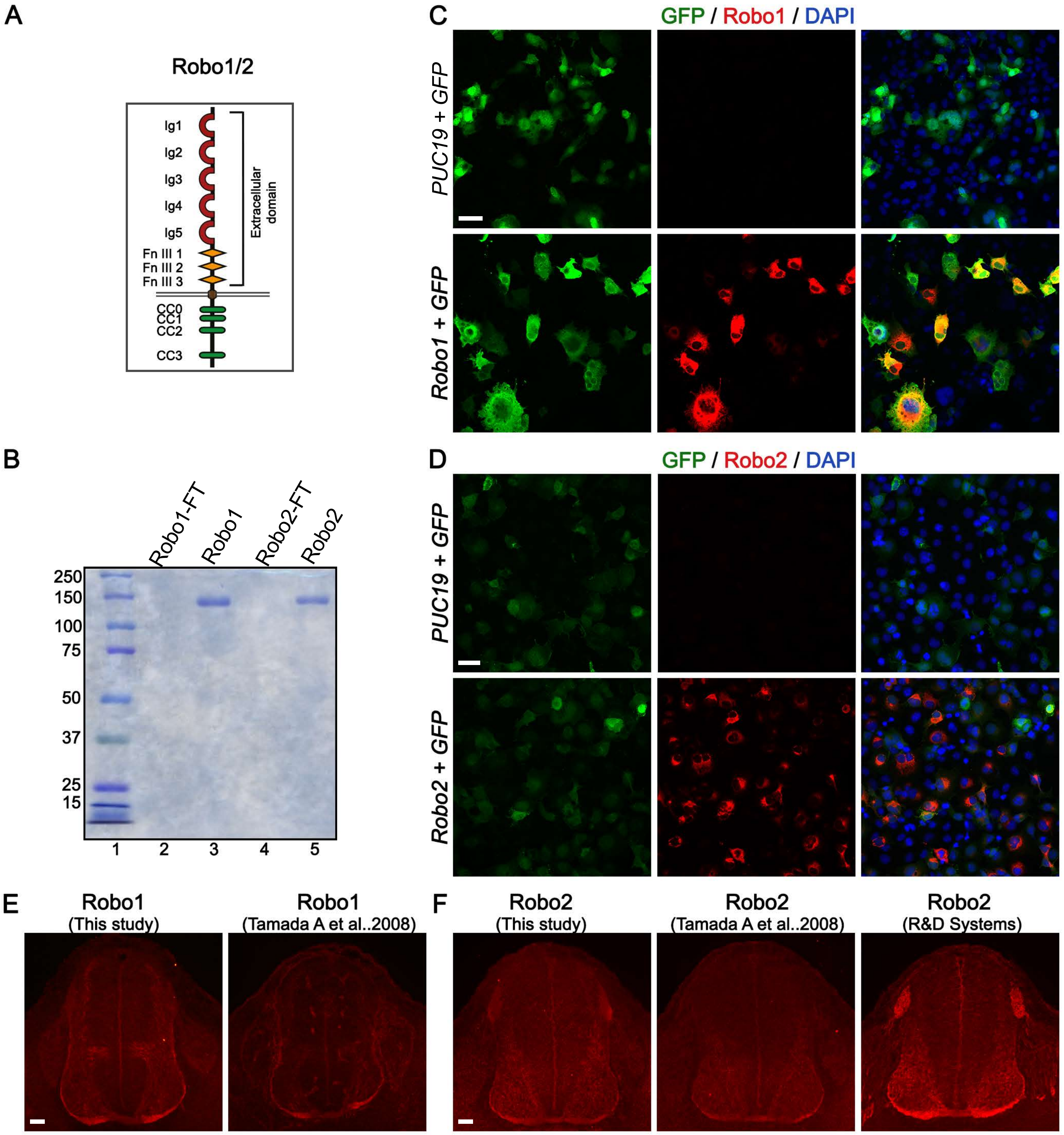

A
Robo1/2
Ig1
Ig2
Ig3
Ig4
Ig5
Fn III 1
Fn III 2
Fn III 3
CC0
CC1
CC2
CC3
Extracellular domain
B
Robo1-FT
Robo1
Robo2-FT
Robo2
250
150
100
75
50
37
25
15
1
2
3
4
5
C
GFP / Robo1 / DAPI
PUC19 + GFP
Robo1 + GFP
D
GFP / Robo2 / DAPI
PUC19 + GFP
Robo2 + GFP
E
Robo1
(This study)
Robo1
(Tamada A et al..2008)
F
Robo2
(This study)
Robo2
(Tamada A et al..2008)
Robo2
(R&D Systems)

Supplementary Figure 1: Generation of the Robo1 and Robo2 antibodies. (**A**) Schematic representation of the domain structure of Robo1 and Robo2 is shown. (**B**) Coomassie blue stained polyacrylamide gel with recombinant Robo1 and Robo2 proteins used for animal inoculation. Lanes 1 – 5 are as follows: Kaleidoscope protein markers (Biorad) (1), flow thorough from the purification and before Robo1 elution (2), purified Robo1 protein used for the inoculation (3), flow thorough from the purification and before Robo2 elution (4), purified Robo2 protein used for the inoculation (5). (**C - D**) COS7 cells transfected with plasmids expressing either GFP (*CMV-GFP/pEGFP-N2*) and control (*PUC19* or *pCDNA3.1*) plasmid or GFP (*CMV-GFP*) and Robo1 ectodomain (*EctoRobo1eFc/pCAG3*) or GFP (*pEGFP-N2*) and Robo2 ectodomain (*EctoRobo2eFc/pCAG3*). The samples were labeled with antibodies against GFP (green) and either Robo1 or Robo2 (red) antibodies made in this study. 3 technical repeats were performed, representative images are shown. (**E - F**) Photomicrographs of mouse embryonic spinal cord tissue transverse sections (E11.5) immunohistochemically labeled with: (**E**) Robo1 antibody serum from this study (1/1000) compared with a previously validated Robo1 antibody from Tamada *et. al.* (Tamada et al., 2008). (**F**) Robo2 antibody serum from this study (1/200) compared with a previously validated Robo2 antibody from Tamada *et. al.*(Tamada et al., 2008) and Robo2 R&D Systems (1:100). Robo2 antibodies were incubated overnight at 30°C in blocking buffer containing 5% FBS, 0.1% Triton X 100 and 1X PBS (77mM Na2HPO4, 23mM NaH2PO4, 1.5M NaCl). At least 3 embryos for each condition were analyzed. Representative images are shown. Scale bars are 50 μm and represent all images in **C, D, E** and **F** respectively.

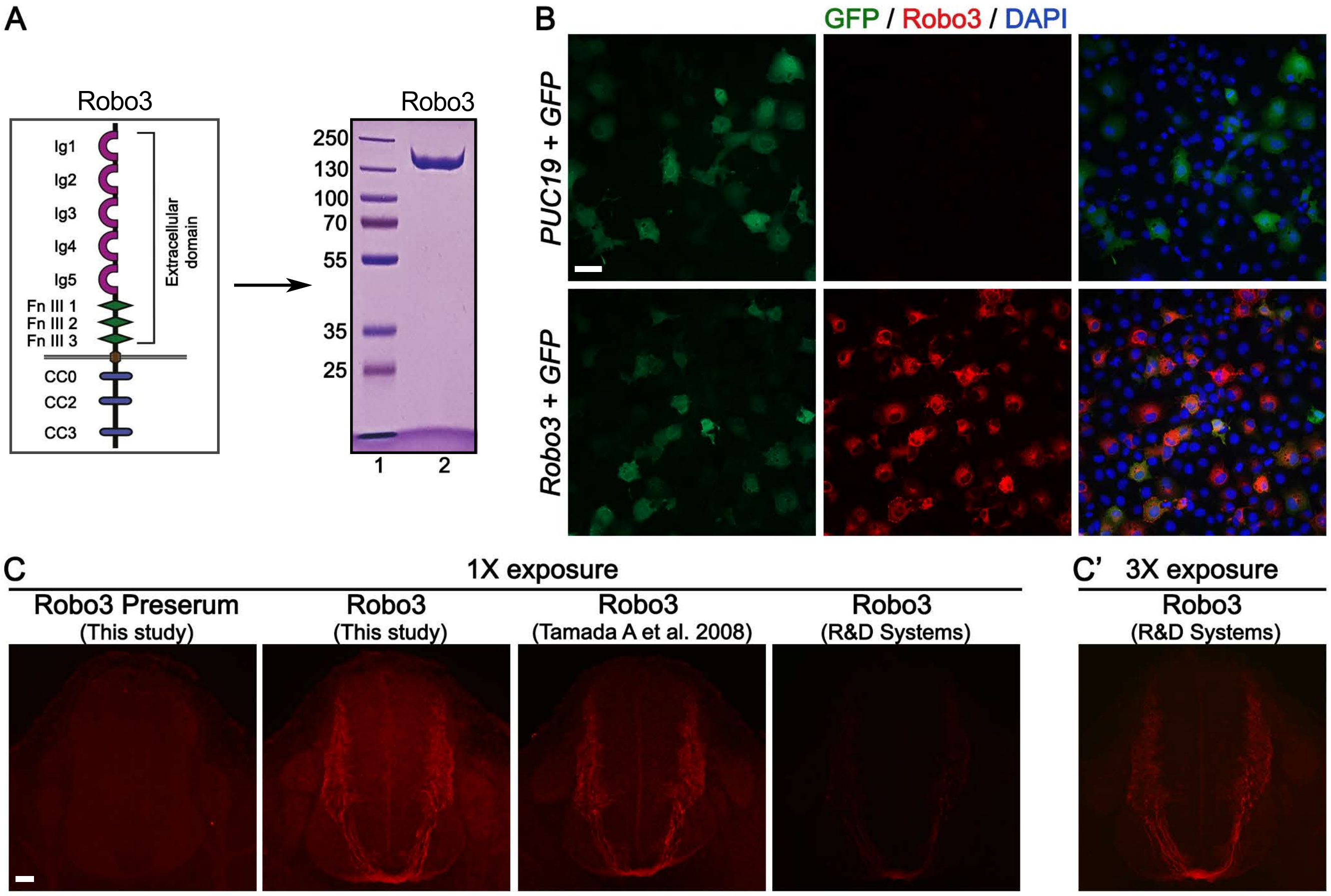
A
Robo3
Ig1
Ig2
Ig3
Ig4
Ig5
Fn III 1
Fn III 2
Fn III 3
CC0
CC2
CC3
Extracellular domain
Robo3
250
130
100
70
55
35
25
1
2
B
GFP / Robo3 / DAPI
PUC19 + GFP
Robo3 + GFP
C
1X exposure
Robo3 Preserum
(This study)
Robo3
(This study)
Robo3
(Tamada A et al. 2008)
Robo3
(R&D Systems)
C'
3X exposure
Robo3
(R&D Systems)

Supplementary Figure 2: Generation of the Robo3 antibody. (**A**) Schematic representation of the domain structure of Robo3 is shown. (**B**) The purified Robo3 protein used for animal inoculation is shown on a Coomassi blue stained polyacrylamide gel. Lanes 1 - 2: Protein Ladder (Thermo Scientific™ 26619) and purified Robo3 protein used for the inoculation. (**C**) COS-7 cells transfected with plasmids expressing either GFP (*pEGFP-N2*) and control plasmid (*PUC19*) or GFP (*pEGFP-N2*) and Robo3 ectodomain (*EctoRig1eFc/pCAG3*) and labeled with antibodies against GFP (green) and Robo3 (red) made in this study. 3 technical repeats were performed, representative images are shown. (**D**) Photomicrographs of mouse embryonic spinal cord tissue sections (E11.5) immunohistochemically labeled with, rabbit pre-immune serum from this study (1/500), α-rabbit Robo3 antibody serum made in this study (1/500), compared with a previously validated α-rabbit Robo3 antibody (1/500) (Tamada et al., 2008) and commercially available α-goat Robo3 antibody (1/500) (R&D Systems). At least 3 embryos for each condition were analyzed. For direct comparison with the antibody made in this study, the exposure time was the same for all images in **D**. Since the antibody from R&D Systems showed substantially weaker labeling, an image with 3x exposure time is also shown. Representative images are shown. Scale bars are 50 µm and represent all images in **C** and **D** respectively.

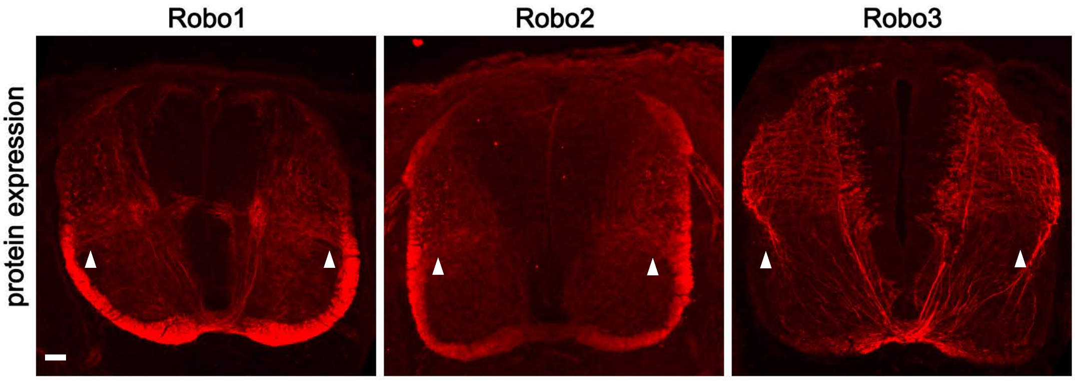
Robo1
Robo2
Robo3
protein expression

Supplementary Figure 3: Photomicrographs of mouse *wild type* (without the *Barhl2*^{GFP} transgene) embryonic brachial spinal cord tissue (E12.5) immunohistochemically labeled with Robo1, Robo2 and Robo3 (red). At least 3 embryos were analyzed for each condition. Representative images are shown. The arrowhead within the deep dorsal horn indicates the position corresponding to dI1 neurons. Scale bar is 50 μm and represents all images.

Figure S4

Barhl2^{GFP} *Math1*^{LacZ}

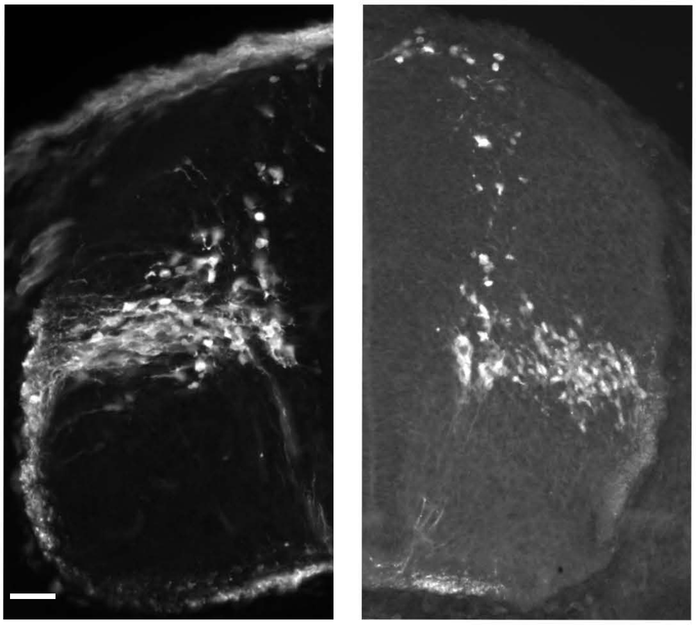

Supplementary Figure 4: *Barhl2*^{GFP} and *Math1*^{LacZ} transgenes delineate dI1 neurons. Photomicrographs of brachial spinal cord sections from E12.5 *Barhl2*^{GFP} (n = 6) and *Math1*^{LacZ} (n = 6) embryos. The images present immunohistochemical labeling with GFP (left) or β-galactosidase (right) of *Barhl2*^{GFP} and *Math1*^{LacZ} embryos respectively. Labeling varies depending on age and axial level and equivalent age and axial level is depicted in the Figure. Scale bar is 50 μm

Figure S5

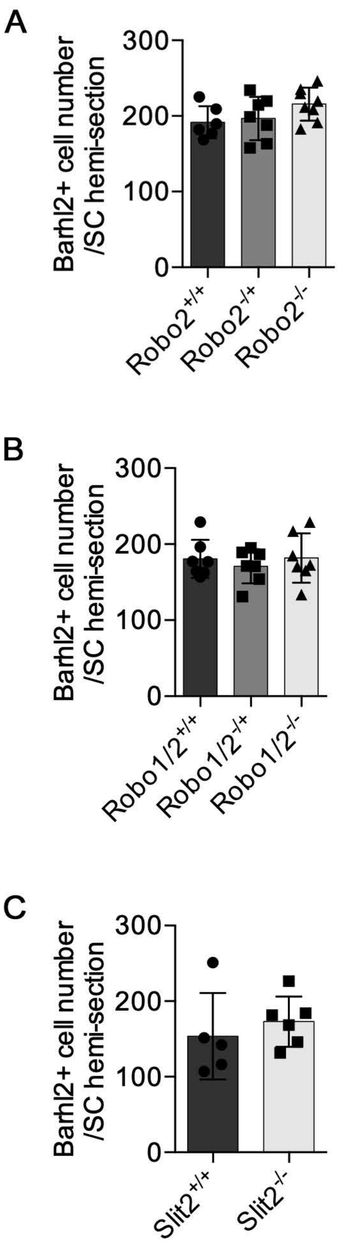
A
Barhl2+ cell number
/SC hemi-section
300
200
100
0
Robo2+/+
Robo2-/+
Robo2-/-
B
Barhl2+ cell number
/SC hemi-section
300
200
100
0
Robo1/2+/+
Robo1/2-/+
Robo1/2-/-
C
Barhl2+ cell number
/SC hemi-section
300
200
100
0
Slit2+/+
Slit2-/-

Supplementary Figure 5: Quantification of Barhl2$^+$ cell number in mutant and control embryos in Supplementary Tables 1 – 3. (**A – C**) Quantification of the number of Barhl2$^+$ cell number per spinal cord (SC) hemi-section of control *Barhl2^{GFP}* (n = 6), heterozygotes *Robo2$^{-/+}$:Barhl2^{GFP}* (n = 7) and *Robo2* mutant *Robo2$^{-/-}$:Barhl2^{GFP}* (n = 8) embryos from **Supplementary Table 1** (**A**), control *Barhl2^{GFP}* (n = 7), heterozygotes *Robo1/2$^{-/+}$:Barhl2^{GFP}* (n = 7) and *Robo1/2* mutant *Robo1/2$^{-/-}$:Barhl2^{GFP}* (n = 7) embryos from **Supplementary Table 3** (**B**), and control *Math1^{LacZ}* (n = 5) and *Slit2* mutant *Slit2$^{-/-}$: Math1^{LacZ}* (n = 6) from **Supplementary Table 2** (**C**) embryos at E12.5, see **Figure 2** and **Supplementary Figures 9** and **11** for example photomicrographs of the immunolabeled tissues. One way ANOVA Kruskal-Wallis test followed by Dune's multiple comparison analysis was performed to compare more than two groups, Mann-Whitney analysis was performed to compare two groups. No significant difference was found. Spinal cord is abbreviated as S.C.

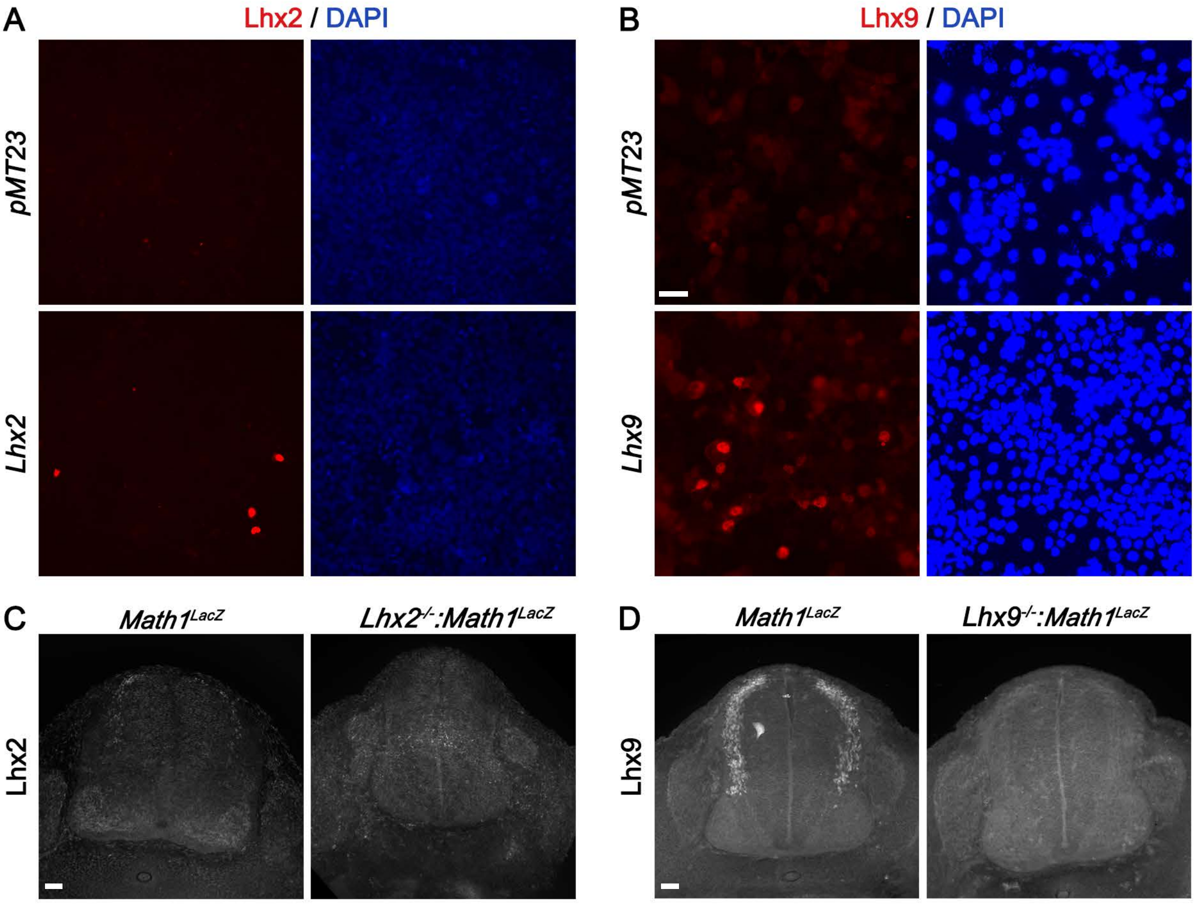
A
Lhx2 / DAPI
pMT23
Lhx2
B
Lhx9 / DAPI
pMT23
Lhx9
C
Math1LacZ
Lhx2-/-:Math1LacZ
Lhx2
D
Math1LacZ
Lhx9-/-:Math1LacZ
Lhx9

Supplementary Figure 6: Lhx2 and Lhx9 antibody generation. (**A - B**) Cell transfections. HEK293 cells were transfected with plasmids expressing control plasmid (*pMT23*) or *Lhx2* expressing plasmid (**A**). COS-7 cells were transfected with plasmids expressing *GFP* expressing plasmid (*CMV-GFP/pEGFP-N2*) and control plasmid (*pMT23*) or *GFP* expressing plasmid (*CMV-GFP/pEGFP-N2*) and *Lhx9* expressing plasmid (**B**). The transfected cells were labeled with antibodies against either rabbit Lhx2 or guinea pig Lhx9 (red) antibodies made in this study and nuclei were counterstained with DAPI. 2 technical repeats were performed for the Lhx9 experiment, and 1 technical repeat was performed for the Lhx2 experiment. Representative images are shown. (**C - D**) E11.5 embryos were generated by crossing $Lhx2^{+/-}$: $Lhx9^{+/-}$: $Math1^{LacZ}$ embryos. The embryos referred to are the following genotypes: *Lhx2* control ($Math1^{LacZ}$, $Lhx2^{+/+}$: $Lhx9^{+/+}$, $Lhx2^{+/+}$: $Lhx9^{+/-}$ or $Lhx2^{+/+}$: $Lhx9^{+/-}$: $Math1^{LacZ}$), *Lhx2* mutant ($Lhx2^{-/-}$: $Lhx9^{+/+}$: $Math1^{LacZ}$, $Lhx2^{-/-}$: $Lhx9^{+/-}$: $Math1^{LacZ}$ or $Lhx2^{-/-}$: $Lhx9^{-/-}$: $Math1^{LacZ}$), *Lhx9* control ($Lhx2^{+/+}$: $Lhx9^{+/+}$: $Math1^{LacZ}$, $Lhx2^{-/-}$: $Lhx9^{+/+}$: $Math1^{LacZ}$; $Lhx2^{-/-}$: $Lhx9^{+/-}$: $Math1^{LacZ}$, $Lhx2^{+/-}$: $Lhx9^{+/-}$: $Math1^{LacZ}$) and *Lhx9* mutant ($Lhx2^{-/-}$: *Lhx9-/-*: $Math1^{LacZ}$ or $Lhx2^{+/-}$: $Lhx9^{-/-}$: $Math1^{LacZ}$). Photomicrographs of transvers sections of *Lhx2* control (n = 5), *Lhx2* mutant (n = 4) or *Lhx9* control (n = 5) or *Lhx9* mutant (n = 4) immunohistochemically labeled with Lhx2 (**C**) or Lhx9 (**D**) are shown. Scale bars are 50 μm.

Figure S7

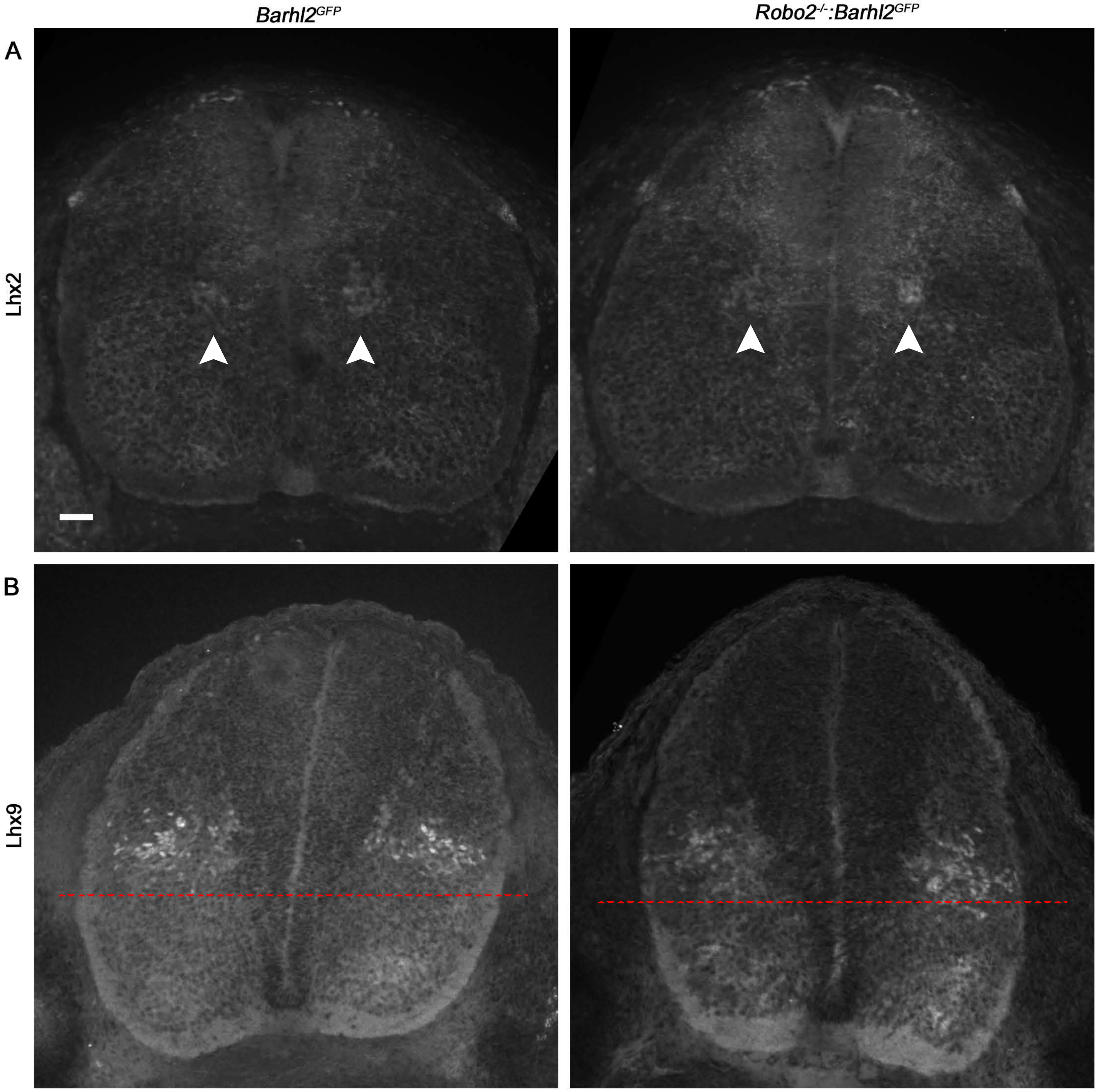

Supplementary Figure 7: Lhx2 and Lhx9 immunolabeling of control and *Robo2* mutant embryos. (**A – B)** Photomicrographs of $Barhl2^{GFP}$ control (Lhx2 labeling n = 3, Lhx9 labeling n = 7) and $Robo2^{-/-}$:$Barhl2^{GFP}$ (Lhx2 labeling n = 3, Lhx9 labeling n = 10) E12.5 mouse embryonic spinal cord brachial (**A**) or thoracic (**B**) transverse sections. The images show immunohistochemical labeling with Lhx2 (**A**) or Lhx9 (**B**) antibodies respectively. The white arrows point to dI1c neurons. The red line represents the delimitation of the ventral cut off of the spinal cord, used to compare the dorso-ventral position of the $Lhx9^+$ cells (38% of the spinal cord dorso-ventral height). (**C**) Quantification of $Lhx9^+$ neurons below a ventral cut off point in *Robo2* mutant (n = 10) and control (n = 7) embryos. Mann-Whitney statistical analysis was used to compare groups, * represents $p < 0.05$. Representative images are shown. Scale bars in **A** is 50 µm and corresponds to **A** and **B**.

Figure S8

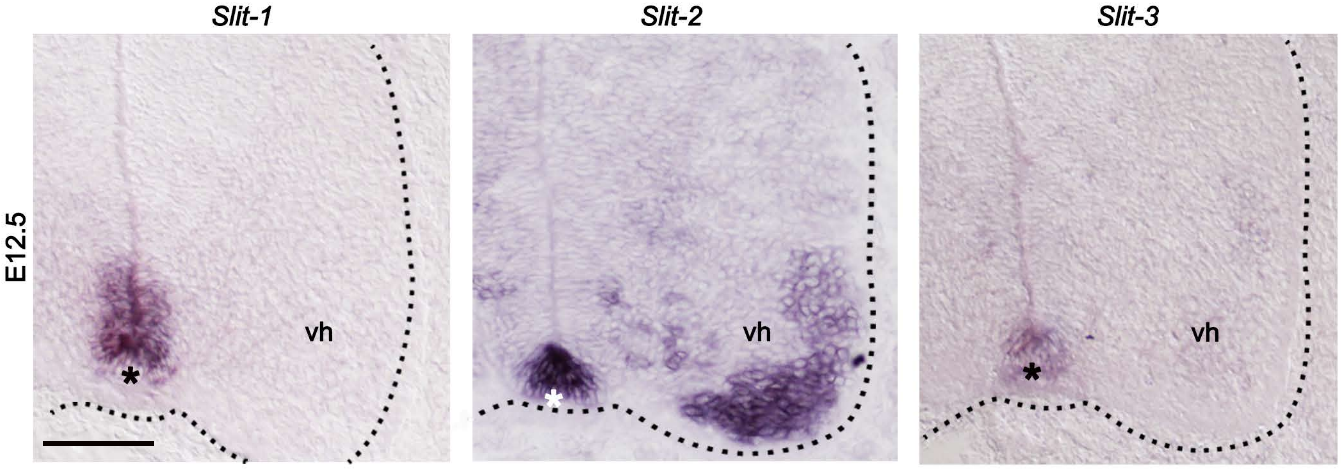

Supplementary Figure 8: *Slit1*, *Slit2*, *Slit3* mRNA distribution in E12.5 mouse spinal cord. Photomicrographs of E12.5 mouse embryo transverse brachial spinal cord sections. Samples were labeled by *in situ* hybridization with DIG-labeled riboprobes against *Slit1, Slit2* and *Slit3*. At least 3 embryos were analyzed for each group. Representative images are shown. The border of the gray matter of the spinal cord is delineated with a black dotted line. The positions of the ventral horn (vh) and floorplate (*) are indicated. The scale bar is 100 µm and represents all images.

Figure S9

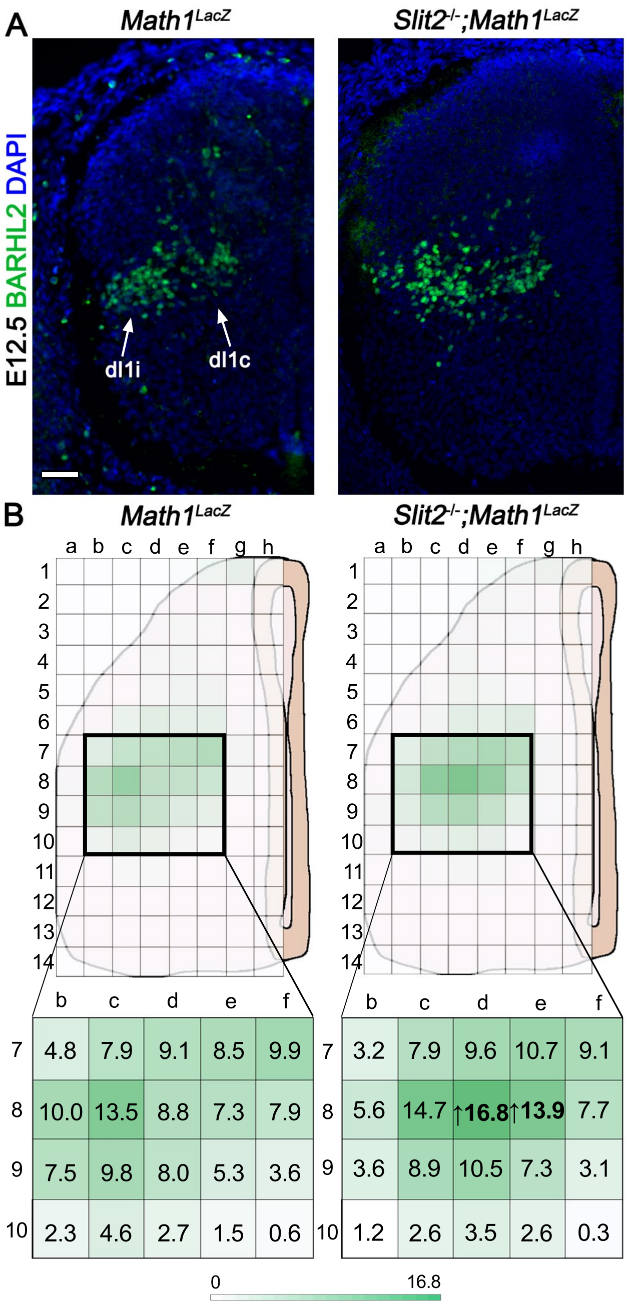
A
Math1LacZ
Slit2-/-;Math1LacZ
E12.5 BARHL2 DAPI
dI1i
dI1c
B
Math1LacZ
Slit2-/-;Math1LacZ
a b c d e f g h
1 2 3 4 5 6 7 8 9 10 11 12 13 14
b c d e f
7 4.8 7.9 9.1 8.5 9.9
8 10.0 13.5 8.8 7.3 7.9
9 7.5 9.8 8.0 5.3 3.6
10 2.3 4.6 2.7 1.5 0.6
b c d e f
7 3.2 7.9 9.6 10.7 9.1
8 5.6 14.7 ↑16.8 ↑13.9 7.7
9 3.6 8.9 10.5 7.3 3.1
10 1.2 2.6 3.5 2.6 0.3
0
16.8

Supplementary Figure 9: Quantification of dI1 cell body migration in *Slit2*$^{-/-}$*:Math1*^{LacZ} and control embryos. (**A**) Example photomicrographs of mouse embryonic spinal cord tissue at E12.5 immunohistochemically labeled with the dI1 transcription factor Barhl2 in control *Math1*^{LacZ} (n = 5) and *Slit2* mutant *Slit2*$^{-/-}$*:Math1*^{LacZ} (n = 6) embryos. (**B**) Schematics representing a E12.5 spinal cord hemi section with the overlying grid used for demarking bins for counting Barhl2^{+} nuclei for quantification are depicted. The Barhl2^{+} mean cell number shown for control (n = 5) and in *Slit2* mutant (n = 6) embryos is depicted by a heat map (green). The bold boxed regions are enlarged. The numbers are the mean number of Barhl2^{+} neurons per bin and the numbers in bold are where statistically significant increase (up arrow) or decrease (down arrow) were found after two-way ANOVA statistical analysis. Each individual box mean and statistical significance are shown in **Supplementary Table 2**. Scale bar in **A** is 50 µm and represents both genotypes.

Figure S10

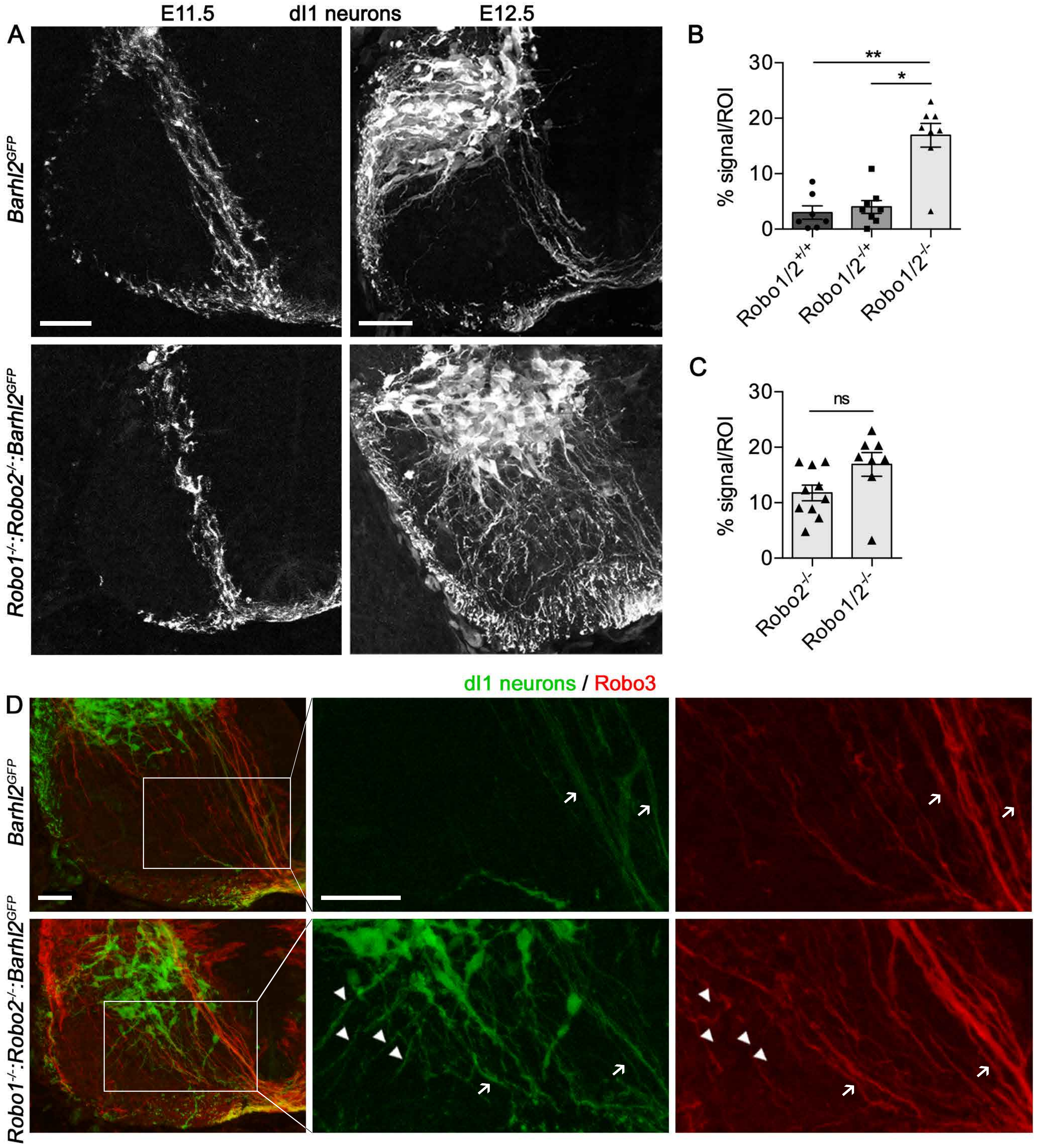

A
E11.5
dI1 neurons
E12.5
Barhl2GFP
Robo1-/-:Robo2-/-:Barhl2GFP
B
% signal/ROI
**
*
Robo1/2+/+
Robo1/2-/+
Robo1/2-/-
C
% signal/ROI
ns
Robo2-/-
Robo1/2-/-
D
dI1 neurons / Robo3
Barhl2GFP
Robo1-/-:Robo2-/-:Barhl2GFP

Supplementary Figure 10: dI1 neurons misproject in *Robo1$^{-/-}$:Robo2$^{-/-}$:Barhl2^{GFP}* embryos. **(A)** Photomicrographs of mouse E11.5 and E12.5 embryonic spinal cord tissue immunohistochemically labeled with GFP (expressed in dI1 neurons, white) in control *Barhl2^{GFP}* (E11.5 n = 3, E12.5 n >8) and *Robo1/2* double mutant *Robo1$^{-/-}$:Robo2$^{-/-}$:Barhl2^{GFP}* (E11.5 n = 3, E12.5 n >8) embryos. Representative images are shown. (**B - C**) Quantification of the GFP$^+$ axons misprojecting into the ventral horn is shown. GFP$^+$ axons pixel intensity in the ventral horn averaged (mean) was measured for all genotypic groups *Barhl2^{GFP}* (n = 8 embryos), *heterozygote Robo1$^{+/-}$:Robo2$^{+/-}$:Barhl2^{GFP}* (n = 8 embryos) and *mutant Robo1$^{-/-}$:Robo2$^{-/-}$:Barhl2^{GFP}* (n = 8 embryos). This measurement is referred to on the graph as % signal per region of interest (ROI). In (**C**) the quantification for *Robo1/2* double mutant embryos and *Robo2* mutant embryos (data from **Figure 3**) are compared. Standard errors and statistical significance are shown. n.s. (not significant) and **** represents $p < 0.0001$ respectively after Kruskal-Wallis statistical analysis. (**D**) Photomicrographs of control *Barhl2^{GFP}* and *mutant Robo1$^{-/-}$:Robo2$^{-/-}$:Barhl2^{GFP}* embryonic spinal cord tissue (E12.5) immunohistochemically labeled with Robo3 (expressed in commissural neurons, red) and GFP (expressed in dI1c and dI1i neurons, green). The boxed areas are enlarged in the single channel images. White arrows point to GFP$^+$/Robo3$^+$ dI1c neurons. White arrow heads point to GFP$^+$/Robo3$^-$ misprojecting dI1i neurons. Representative images are shown. Scale bars in **A** and **D** are 50 μm and represents the scale for corresponding genotypes and ages.

Figure S11

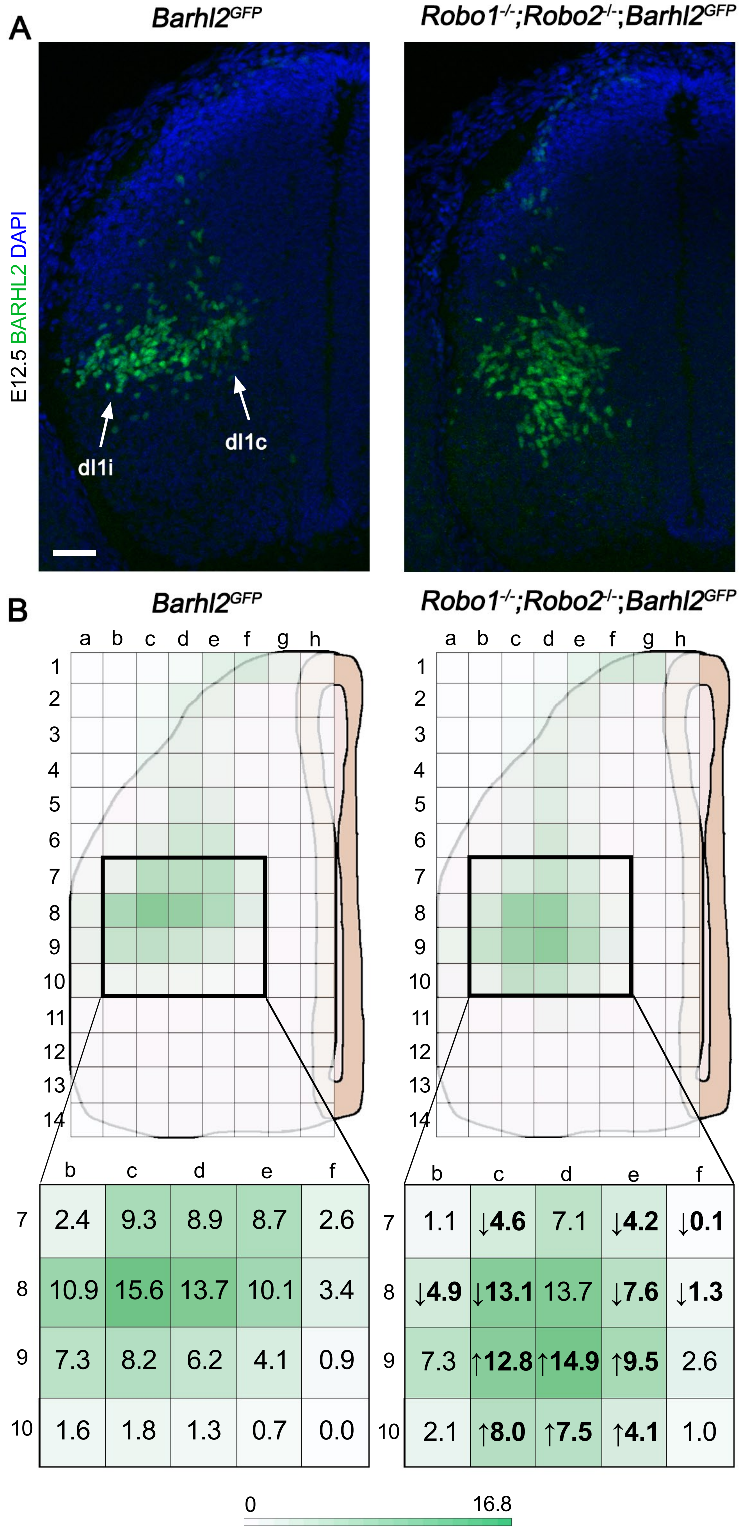

*Barhl2*GFP

	b	c	d	e	f
7	2.4	9.3	8.9	8.7	2.6
8	10.9	15.6	13.7	10.1	3.4
9	7.3	8.2	6.2	4.1	0.9
10	1.6	1.8	1.3	0.7	0.0

Robo1-/-;*Robo2*-/-;*Barhl2*GFP

	b	c	d	e	f
7	1.1	↓**4.6**	7.1	↓**4.2**	↓**0.1**
8	↓**4.9**	↓**13.1**	13.7	↓**7.6**	↓**1.3**
9	7.3	↑**12.8**	↑**14.9**	↑**9.5**	2.6
10	2.1	↑**8.0**	↑**7.5**	↑**4.1**	1.0

Supplementary Figure 11: dI1 cell bodies are misguided in *Robo1$^{-/-}$:Robo2$^{-/-}$:Barhl2^{GFP}* embryos. (**A**) Example photomicrographs of control *Barhl2^{GFP}* and double mutant *Robo1$^{-/-}$:Robo2$^{-/-}$:Barhl2^{GFP}* embryonic spinal cord tissue (E12.5) immunohistochemically labeled with the dI1 transcription factor Barhl2. (**B**) Schematics representing a E12.5 spinal cord hemi section with the overlying grid used for demarking bins for counting Barhl2^{+} nuclei for quantification are depicted. The mean number of Barhl2^{+} cell number shown for control *Barhl2^{GFP}* (n = 7 embryos) and *mutant Robo1$^{-/-}$:Robo2$^{-/-}$:Barhl2^{GFP}* (n = 7 embryos) embryos is depicted by a heat map (green). The bold boxed regions are enlarged to show the mean number of Barhl2^{+} neurons per bin, values in bold are where statistically significant increase (up arrow) or decrease (down arrow) after two-way ANOVA statistical analysis. Each individual box mean and statistical significance are shown in **Supplementary Table 3**. Scale bar in **A** is 50 µm and represents both genotypes.

2.2 Supplementary Tables

Supplementary Table 1: Mean and statistical significance for Barhl2^{+} neurons in E12.5 spinal cord sections of *Robo2* mutant and control embryos. The data are the extended data from **Figure 2**. The number of embryos used in this analysis were *Robo2* mutant (n = 8), *Robo2* heterozygote (n = 7) and control (n= 6) embryos. Significance is indicated by *, **, *** or **** respectively representing pvalue < 0.05, 0.01, 0.001, 0.0001 from ANOVA followed by Sidak multiple comparison tests.

	Average cell/bin			ANOVA		
	Robo2+/+	Robo2-/+	Robo2-/-	WT vs. Robo2$^{-/+}$	Robo2$^{-/+}$ vs. Robo2$^{-/-}$	WT vs. Robo2$^{-/-}$
A1	0.00	0.00	0.00	ns	ns	ns
B1	0.00	0.00	0.00	ns	ns	ns
C1	0.00	0.43	0.00	ns	ns	ns
D1	0.10	1.00	0.69	ns	ns	ns
E1	0.40	1.07	2.27	ns	ns	ns
F1	1.70	1.29	1.56	ns	ns	ns
G1	1.30	1.43	0.25	ns	ns	ns
H1	0.00	0.07	0.06	ns	ns	ns
A2	0.00	0.00	0.00	ns	ns	ns
B2	0.00	0.00	0.00	ns	ns	ns
C2	0.30	1.14	1.13	ns	ns	ns
D2	0.80	0.71	1.56	ns	ns	ns
E2	1.30	1.50	1.00	ns	ns	ns
F2	0.60	0.71	0.00	ns	ns	ns
G2	0.10	0.00	0.00	ns	ns	ns
H2	0.00	0.00	0.00	ns	ns	ns
A3	0.00	0.00	0.00	ns	ns	ns
B3	0.00	0.57	0.00	ns	ns	ns
C3	0.80	1.57	2.13	ns	ns	ns
D3	1.20	0.71	1.38	ns	ns	ns
E3	2.20	1.86	1.13	ns	ns	ns
F3	0.20	0.36	0.08	ns	ns	ns
G3	0.00	0.00	0.00	ns	ns	ns
H3	0.00	0.00	0.00	ns	ns	ns
A4	0.00	0.00	0.00	ns	ns	ns
B4	0.00	0.07	0.25	ns	ns	ns
C4	0.40	0.93	2.19	ns	ns	ns
D4	2.10	1.07	1.69	ns	ns	ns
E4	1.20	1.43	1.50	ns	ns	ns
F4	0.50	0.50	0.00	ns	ns	ns

G4	0.00	0.00	0.00	ns	ns	ns
H4	0.00	0.00	0.00	ns	ns	ns
A5	0.00	0.00	0.00	ns	ns	ns
B5	0.00	0.00	0.69	ns	ns	ns
C5	0.30	0.57	2.29	ns	ns	ns
D5	1.80	1.86	2.85	ns	ns	ns
E5	2.20	2.50	1.54	ns	ns	ns
F5	0.90	1.14	0.31	ns	ns	ns
G5	0.20	0.00	0.00	ns	ns	ns
H5	0.10	0.00	0.00	ns	ns	ns
A6	0.00	0.00	0.00	ns	ns	ns
B6	0.60	0.07	0.31	ns	ns	ns
C6	2.50	1.43	2.73	ns	ns	ns
D6	5.40	4.93	5.48	ns	ns	ns
E6	4.80	3.64	4.00	ns	ns	ns
F6	2.60	1.64	1.75	ns	ns	ns
G6	0.30	0.00	0.04	ns	ns	ns
H6	0.00	0.00	0.00	ns	ns	ns
A7	1.00	0.43	0.00	ns	ns	ns
B7	2.70	1.71	0.77	ns	*	ns
C7	6.40	6.14	6.48	ns	ns	ns
D7	10.30	9.00	11.98	ns	ns	*
E7	9.50	9.64	10.08	ns	ns	ns
F7	5.70	3.79	4.10	**	*	ns
G7	0.10	0.21	0.00	ns	ns	ns
H7	0.00	0.00	0.00	ns	ns	ns
A8	1.80	2.00	0.69	ns	ns	ns
B8	10.30	15.29	4.23	****	****	****
C8	15.60	19.21	13.52	****	ns	****
D8	13.60	17.57	14.81	****	*	*
E8	11.90	13.43	16.58	***	****	ns
F8	4.30	5.79	6.13	ns	ns	ns
G8	0.00	0.36	0.00	ns	ns	ns
H8	0.00	0.00	0.00	ns	ns	ns
A9	1.00	2.00	0.56	ns	ns	ns
B9	8.50	11.07	4.21	***	**	****
C9	12.30	13.29	10.52	ns	ns	*
D9	8.70	11.71	16.15	**	****	**
E9	6.60	6.57	14.31	ns	****	****
F9	0.50	1.79	4.35	ns	ns	ns
G9	0.00	0.14	0.33	ns	ns	ns
H9	0.00	0.00	0.00	ns	ns	ns

A10	0.90	0.86	0.44	ns	ns	ns
B10	5.20	1.07	0.88	*	**	ns
C10	3.00	1.71	3.52	ns	ns	ns
D10	2.80	2.86	10.35	ns	****	****
E10	2.80	1.64	8.27	ns	****	****
F10	0.20	0.14	2.21	ns	ns	ns
G10	0.00	0.07	0.21	ns	ns	ns
H10	0.00	0.00	0.00	ns	ns	ns
A11	0.20	0.00	0.00	ns	ns	ns
B11	1.00	0.07	0.00	ns	ns	ns
C11	0.50	0.21	0.94	ns	ns	ns
D11	0.30	0.29	2.38	ns	ns	ns
E11	0.20	0.36	1.94	ns	ns	ns
F11	0.10	0.00	0.63	ns	ns	ns
G11	0.10	0.00	0.00	ns	ns	ns
H11	0.00	0.00	0.00	ns	ns	ns
A12	0.00	0.00	0.00	ns	ns	ns
B12	0.00	0.00	0.00	ns	ns	ns
C12	0.00	0.00	0.00	ns	ns	ns
D12	0.10	0.00	0.27	ns	ns	ns
E12	0.00	0.00	0.56	ns	ns	ns
F12	0.00	0.00	0.17	ns	ns	ns
G12	0.00	0.00	0.00	ns	ns	ns
H12	0.00	0.00	0.00	ns	ns	ns
A13	0.00	0.00	0.00	ns	ns	ns
B13	0.00	0.00	0.00	ns	ns	ns
C13	0.00	0.00	0.00	ns	ns	ns
D13	0.10	0.00	0.06	ns	ns	ns
E13	0.00	0.00	0.13	ns	ns	ns
F13	0.00	0.00	0.00	ns	ns	ns
G13	0.00	0.00	0.00	ns	ns	ns
H13	0.00	0.00	0.00	ns	ns	ns
A14	0.00	0.00	0.00	ns	ns	ns
B14	0.00	0.00	0.00	ns	ns	ns
C14	0.00	0.00	0.00	ns	ns	ns
D14	0.00	0.00	0.00	ns	ns	ns
E14	0.00	0.00	0.00	ns	ns	ns
F14	0.00	0.00	0.00	ns	ns	ns
G14	0.00	0.00	0.00	ns	ns	ns
H14	0.00	0.00	0.00	ns	ns	ns

Supplementary Table 2: Mean and statistical significance for Barhl2^{+} neurons in E12.5 spinal cord sections of *Slit2* mutant and control embryos. The data are the extended data from **Figure S9**. The number of embryos used in this analysis were *Slit2* mutant (n = 6) and control (n= 5) embryos. Significance is indicated by *, **, *** or **** respectively representing pvalue < 0.05, 0.01, 0.001, 0.0001 from ANOVA followed by Sidak multiple comparison tests.

	Average cell/bin		ANOVA
	Slit2+/+	Slit2-/-	Slit2+/+ vs Slit2-/-
A1	0.00	0.00	ns
B1	0.00	0.00	ns
C1	0.00	0.00	ns
D1	0.00	0.25	ns
E1	0.63	1.08	ns
F1	1.88	1.33	ns
G1	3.25	1.92	ns
H1	0.63	1.00	ns
A2	0.00	0.00	ns
B2	0.00	0.00	ns
C2	0.00	0.00	ns
D2	0.00	0.42	ns
E2	0.38	0.92	ns
F2	0.38	0.50	ns
G2	0.00	0.17	ns
H2	0.00	0.00	ns
A3	0.00	0.00	ns
B3	0.00	0.00	ns
C3	0.00	0.08	ns
D3	0.38	0.75	ns
E3	0.75	0.42	ns
F3	0.25	0.25	ns
G3	0.00	0.00	ns
H3	0.00	0.00	ns
A4	0.00	0.00	ns
B4	0.00	0.00	ns
C4	0.00	0.08	ns
D4	0.88	1.08	ns
E4	0.75	0.17	ns
F4	0.13	0.08	ns
G4	0.00	0.00	ns
H4	0.00	0.00	ns

A5	0.00	0.00	ns
B5	0.13	0.00	ns
C5	0.13	0.42	ns
D5	0.88	1.75	ns
E5	1.63	1.08	ns
F5	1.00	0.17	ns
G5	0.00	0.00	ns
H5	0.00	0.00	ns
A6	0.00	0.00	ns
B6	0.38	0.17	ns
C6	2.13	1.25	ns
D6	3.75	3.42	ns
E6	3.00	2.92	ns
F6	2.63	3.92	ns
G6	0.38	0.00	ns
H6	0.00	0.00	ns
A7	0.25	0.08	ns
B7	4.75	3.25	ns
C7	7.88	7.92	ns
D7	9.13	9.58	ns
E7	8.50	10.75	ns
F7	9.88	9.08	ns
G7	1.50	0.83	ns
H7	0.00	0.00	ns
A8	0.63	0.08	ns
B8	10.00	5.58	ns
C8	13.50	14.67	ns
D8	8.75	16.83	****
E8	7.25	13.92	**
F8	7.88	7.67	ns
G8	1.13	0.67	ns
H8	0.00	0.00	ns
A9	0.50	0.83	ns
B9	7.50	3.58	ns
C9	9.75	8.92	ns
D9	8.00	10.50	ns
E9	5.25	7.33	ns
F9	3.63	3.08	ns
G9	0.13	0.25	ns
H9	0.00	0.00	ns
A10	0.13	0.25	ns
B10	2.25	1.25	ns

C10	4.63	2.58	ns
D10	2.75	3.50	ns
E10	1.50	2.58	ns
F10	0.63	0.33	ns
G10	0.00	0.00	ns
H10	0.00	0.00	ns
A11	0.00	0.00	ns
B11	0.00	0.00	ns
C11	0.75	0.33	ns
D11	0.63	0.92	ns
E11	0.00	0.67	ns
F11	0.00	0.00	ns
G11	0.00	0.08	ns
H11	0.00	0.00	ns
A12	0.00	0.00	ns
B12	0.00	0.00	ns
C12	0.00	0.00	ns
D12	0.00	0.00	ns
E12	0.00	0.00	ns
F12	0.00	0.00	ns
G12	0.00	0.00	ns
H12	0.00	0.00	ns
A13	0.00	0.00	ns
B13	0.00	0.00	ns
C13	0.00	0.00	ns
D13	0.00	0.00	ns
E13	0.00	0.00	ns
F13	0.00	0.00	ns
G13	0.00	0.00	ns
H13	0.00	0.00	ns
A14	0.00	0.00	ns
B14	0.00	0.00	ns
C14	0.00	0.00	ns
D14	0.00	0.00	ns
E14	0.00	0.00	ns
F14	0.00	0.00	ns
G14	0.00	0.00	ns
H14	0.00	0.00	ns

Supplementary Table 3: Mean and statistical significance for Barhl2^{+} neurons in E12.5 spinal cord sections of *Robo1/Robo2* mutant and control embryos. The data are the extended data from **Figure S11**. The number of embryos used in this analysis were *Barhl2^{GFP}* (n = 7 embryos), *heterozygote Robo1$^{+/-}$:Robo2$^{+/-}$:Barhl2^{GFP}* (n = 7 embryos) and *mutant Robo1$^{-/-}$:Robo2$^{-/-}$:Barhl2^{GFP}* (n = 7 embryos). Significance is indicated by *, **, *** or **** respectively representing pvalue < 0.05, 0.01, 0.001, 0.0001 from ANOVA followed by Sidak multiple comparison tests.

	Average cell/bin			ANOVA		
	Robo1+/+ Robo2+/+	Robo1-/+ Robo2-/+	Robo1-/- Robo2-/-	WT vs. Robo1/2$^{-/+}$	Robo1/2$^{-/+}$ vs. Robo1/2$^{-/-}$	WT vs. Robo1/2$^{-/-}$
A1	0.00	0.00	0.00	ns	ns	ns
B1	0.00	0.00	0.00	ns	ns	ns
C1	0.29	0.00	0.00	ns	ns	ns
D1	1.21	1.86	0.57	ns	ns	ns
E1	3.71	4.21	2.86	ns	ns	ns
F1	4.57	3.36	4.93	ns	ns	ns
G1	2.00	1.50	4.86	ns	***	**
H1	0.00	0.07	0.86	ns	ns	ns
A2	0.00	0.00	0.00	ns	ns	ns
B2	0.00	0.00	0.00	ns	ns	ns
C2	0.93	0.93	0.50	ns	ns	ns
D2	3.07	2.43	1.71	ns	ns	ns
E2	2.21	1.14	3.00	ns	ns	ns
F2	0.21	0.07	0.57	ns	ns	ns
G2	0.07	0.00	0.07	ns	ns	ns
H2	0.00	0.00	0.00	ns	ns	ns
A3	0.00	0.00	0.00	ns	ns	ns
B3	0.07	0.00	0.00	ns	ns	ns
C3	1.64	2.36	0.93	ns	ns	ns
D3	2.50	2.93	2.36	ns	ns	ns
E3	0.93	1.29	1.71	ns	ns	ns
F3	0.00	0.00	0.00	ns	ns	ns
G3	0.00	0.00	0.00	ns	ns	ns
H3	0.00	0.00	0.00	ns	ns	ns
A4	0.00	0.00	0.00	ns	ns	ns
B4	0.07	0.21	0.07	ns	ns	ns
C4	1.79	1.36	1.86	ns	ns	ns
D4	2.43	2.86	3.36	ns	ns	ns
E4	1.36	0.57	1.43	ns	ns	ns
F4	0.00	0.00	0.00	ns	ns	ns

G4	0.00	0.00	0.00	ns	ns	ns
H4	0.00	0.00	0.00	ns	ns	ns
A5	0.00	0.00	0.00	ns	ns	ns
B5	0.07	0.21	0.14	ns	ns	ns
C5	0.36	1.93	1.71	ns	ns	ns
D5	4.07	3.00	3.71	ns	ns	ns
E5	2.50	0.07	1.43	**	ns	ns
F5	0.00	0.00	0.00	ns	ns	ns
G5	0.00	0.00	0.00	ns	ns	ns
H5	0.00	0.00	0.00	ns	ns	ns
A6	0.00	0.07	0.00	ns	ns	ns
B6	0.71	0.93	0.14	ns	ns	ns
C6	2.79	2.07	1.71	ns	ns	ns
D6	6.00	5.79	5.50	ns	ns	ns
E6	5.43	2.71	2.21	**	ns	***
F6	0.36	0.21	0.07	ns	ns	ns
G6	0.00	0.00	0.00	ns	ns	ns
H6	0.00	0.00	0.00	ns	ns	ns
A7	0.43	0.14	0.07	ns	ns	ns
B7	2.36	3.07	1.14	ns	*	ns
C7	9.29	7.57	4.57	ns	***	****
D7	8.93	9.64	7.07	ns	**	ns
E7	8.71	6.43	4.21	*	*	****
F7	2.64	1.14	0.14	ns	ns	**
G7	0.00	0.00	0.00	ns	ns	ns
H7	0.00	0.00	0.00	ns	ns	ns
A8	2.21	1.64	0.71	ns	ns	ns
B8	10.86	9.93	4.93	ns	****	****
C8	15.64	14.79	13.07	ns	ns	**
D8	13.71	13.79	13.71	ns	ns	ns
E8	10.07	9.29	7.64	ns	ns	**
F8	3.36	2.00	1.29	ns	ns	*
G8	0.00	0.00	0.00	ns	ns	ns
H8	0.00	0.00	0.00	ns	ns	ns
A9	2.29	1.93	2.07	ns	ns	ns
B9	7.29	8.50	7.29	ns	ns	ns
C9	8.21	11.79	12.79	****	ns	****
D9	6.21	9.64	14.86	****	****	****
E9	4.14	5.43	9.50	ns	****	****
F9	0.93	0.36	2.57	ns	*	ns
G9	0.07	0.00	0.00	ns	ns	ns
H9	0.00	0.00	0.00	ns	ns	ns

A10	1.29	0.71	0.79	ns	ns	ns
B10	1.57	1.14	2.14	ns	ns	ns
C10	1.79	2.29	8.00	ns	****	****
D10	1.29	3.00	7.50	ns	****	****
E10	0.71	0.71	4.14	ns	****	****
F10	0.00	0.36	1.00	ns	ns	ns
G10	0.00	0.00	0.00	ns	ns	ns
H10	0.00	0.00	0.00	ns	ns	ns
A11	0.00	0.00	0.14	ns	ns	ns
B11	0.07	0.14	0.07	ns	ns	ns
C11	0.07	0.50	0.21	ns	ns	ns
D11	0.07	0.50	1.14	ns	ns	ns
E11	0.00	0.21	0.57	ns	ns	ns
F11	0.00	0.00	0.21	ns	ns	ns
G11	0.07	0.00	0.00	ns	ns	ns
H11	0.00	0.00	0.00	ns	ns	ns
A12	0.00	0.00	0.00	ns	ns	ns
B12	0.00	0.07	0.00	ns	ns	ns
C12	0.07	0.07	0.00	ns	ns	ns
D12	0.00	0.14	0.00	ns	ns	ns
E12	0.00	0.07	0.00	ns	ns	ns
F12	0.00	0.00	0.00	ns	ns	ns
G12	0.00	0.00	0.00	ns	ns	ns
H12	0.00	0.00	0.00	ns	ns	ns
A13	0.00	0.00	0.00	ns	ns	ns
B13	0.00	0.00	0.00	ns	ns	ns
C13	0.00	0.00	0.00	ns	ns	ns
D13	0.00	0.00	0.00	ns	ns	ns
E13	0.00	0.00	0.00	ns	ns	ns
F13	0.00	0.00	0.00	ns	ns	ns
G13	0.00	0.00	0.00	ns	ns	ns
H13	0.00	0.00	0.00	ns	ns	ns
A14	0.00	0.00	0.00	ns	ns	ns
B14	0.00	0.00	0.00	ns	ns	ns
C14	0.00	0.00	0.00	ns	ns	ns
D14	0.00	0.00	0.00	ns	ns	ns
E14	0.00	0.00	0.00	ns	ns	ns
F14	0.00	0.00	0.00	ns	ns	ns
G14	0.00	0.00	0.00	ns	ns	ns
H14	0.00	0.00	0.00	ns	ns	ns

3 Supplementary References

Kropp, M. and Wilson, S. I. (2012). The expression profile of the tumor suppressor gene Lzts1 suggests a role in neuronal development. *Dev Dyn* **241**, 984-994.

Tamada, A., Kumada, T., Zhu, Y., Matsumoto, T., Hatanaka, Y., Muguruma, K., Chen, Z., Tanabe, Y., Torigoe, M., Yamauchi, K., et al. (2008). Crucial roles of Robo proteins in midline crossing of cerebellofugal axons and lack of their up-regulation after midline crossing. *Neural Dev* **3**, 29.
